# Blueberry Counteracts Prediabetes in a Hypercaloric Diet-Induced Rat Model and Rescues Hepatic Mitochondrial Bioenergetics

**DOI:** 10.3390/nu13124192

**Published:** 2021-11-23

**Authors:** Sara Nunes, Sofia D. Viana, Inês Preguiça, André Alves, Rosa Fernandes, João S. Teodoro, Patrícia Matos, Artur Figueirinha, Lígia Salgueiro, Alexandra André, Sara Silva, Ivana Jarak, Rui A. Carvalho, Cláudia Cavadas, Anabela P. Rolo, Carlos M. Palmeira, Maria M. Pintado, Flávio Reis

**Affiliations:** 1 Institute of Pharmacology & Experimental Therapeutics & Coimbra Institute for Clinical and Biomedical Research (iCBR), Faculty of Medicine, University of Coimbra, 3000-548 Coimbra, Portugal; spnunes@fmed.uc.pt (S.N.); sofia.viana@uc.pt (S.D.V.); i.preguica@campus.fct.unl.pt (I.P.); alves.andrefb@gmail.com (A.A.); rcfernandes@fmed.uc.pt (R.F.); 2 Center for Innovative Biomedicine and Biotechnology (CIBB), University of Coimbra, 3004-504 Coimbra, Portugal; ccavadas@uc.pt; 3 Clinical Academic Center of Coimbra (CACC), 3004-504 Coimbra, Portugal; 4 Polytechnic Institute of Coimbra, ESTESC-Coimbra Health School, Pharmacy/Biomedical Laboratory Sciences, 3046-854 Coimbra, Portugal; alexandra.andre@estescoimbra.pt; 5 Department of Life Sciences, Faculty of Science and Technology (FCTUC), University of Coimbra, 3000-456 Coimbra, Portugal; jteodoro@ci.uc.pt (J.S.T.); rac@uc.pt (R.A.C.); anpiro@ci.uc.pt (A.P.R.); palmeira@uc.pt (C.M.P.); 6 Center for Neurosciences and Cell Biology of Coimbra (CNC), University of Coimbra, 3004-504 Coimbra, Portugal; 7 Faculty of Pharmacy, University of Coimbra, 3000-548 Coimbra, Portugal; patricia_matos_20@hotmail.com (P.M.); amfigueirinha@ff.uc.pt (A.F.); ligia@ff.uc.pt (L.S.); 8 LAQV, REQUIMTE, Faculty of Pharmacy, University of Coimbra, 3000-456 Coimbra, Portugal; 9 CIEPQPF, Chemical Process Engineering and Forest Products Research Centre Research Center, University of Coimbra, 3000-456 Coimbra, Portugal; 10 CBQF—Centro de Biotecnologia e Química Fina—Laboratório Associado, Escola Superior de Biotecnologia, Universidade Católica Portuguesa, Rua Diogo Botelho 1327, 4169-005 Porto, Portugal; snsilva@porto.ucp.pt (S.S.); mpintado@porto.ucp.pt (M.M.P.); 11 Department of Microscopy, Laboratory of Cell Biology and Unit for Multidisciplinary Research in Biomedicine (UMIB), Institute of Biomedical Sciences Abel Salazar (ICBAS), University of Porto, 4050-313 Porto, Portugal; jarak.ivana@gmail.com; 12 Associated Laboratory for Green Chemistry-Clean Technologies and Processes, REQUIMTE, Faculty of Sciences and Technology, University of Porto, 4050-313 Porto, Portugal

**Keywords:** blueberries, hypercaloric diet-induced prediabetes, gut microbiota, hepatic energy metabolism, mitochondria function, hepatoprotection

## Abstract

The paramount importance of a healthy diet in the prevention of type 2 diabetes is now well recognized. Blueberries (BBs) have been described as attractive functional fruits for this purpose. This study aimed to elucidate the cellular and molecular mechanisms pertaining to the protective impact of blueberry juice (BJ) on prediabetes. Using a hypercaloric diet-induced prediabetic rat model, we evaluated the effects of BJ on glucose, insulin, and lipid profiles; gut microbiota composition; intestinal barrier integrity; and metabolic endotoxemia, as well as on hepatic metabolic surrogates, including several related to mitochondria bioenergetics. BJ supplementation for 14 weeks counteracted diet-evoked metabolic deregulation, improving glucose tolerance, insulin sensitivity, and hypertriglyceridemia, along with systemic and hepatic antioxidant properties, without a significant impact on the gut microbiota composition and related mechanisms. In addition, BJ treatment effectively alleviated hepatic steatosis and mitochondrial dysfunction observed in the prediabetic animals, as suggested by the amelioration of bioenergetics parameters and key targets of inflammation, insulin signaling, ketogenesis, and fatty acids oxidation. In conclusion, the beneficial metabolic impact of BJ in prediabetes may be mainly explained by the rescue of hepatic mitochondrial bioenergetics. These findings pave the way to support the use of BJ in prediabetes to prevent diabetes and its complications.

## 1. Introduction

Prediabetes, characterized by impaired glucose tolerance (IGT) and/or impaired fasting glucose (IFG) [1,2], represents a high-risk state for type 2 diabetes mellitus (T2DM) development. Annually, around 5–10% of prediabetic patients progress to T2DM, and estimates indicate that up to 70% of patients with prediabetes will eventually develop overt diabetes within their lifetime [3,4].

During the last decades, new studies have suggested a chief role for gut microbiota (GM) in the development of metabolic diseases, including T2DM. Although incongruent data subsist, emerging evidence underlines a dysbiotic GM community characterized by changes in bacterial abundance/diversity along with decreased short-chain fatty acids (SCFAs) levels (e.g., butyrate) early in the period of human prediabetes [5,6,7]. Moreover, the liver plays a predominant role in the development of insulin resistance since it is a key organ involved in glucose, lipid, and amino acid metabolism and crucial for maintain energy homeostasis [8]. Notably, both insulin resistance and hepatic glucose/lipid dysmetabolism start years before T2DM diagnosis, underpinning prediabetes’ subclinical evolution [4,9,10,11]. Likewise, hepatic energy dysmetabolism has been traced to mitochondria as early as prediabetes in both rodents [12] and humans [13]. However, a direct causal relationship between insulin resistance and mitochondrial dysfunction still lacks further elucidation [14,15,16]. Collectively, the aforesaid pathophysiological mechanisms often culminate in a state of chronic low-grade inflammation and oxidative stress that follows the progression of prediabetes to overt T2DM [17,18]. In the view of the T2DM health burden, which has reached epidemic proportions [19,20], it is crucial to establish interventions targeted to early pathophysiological mechanisms in the asymptomatic prediabetic phase, an opportunity window to prevent or attenuate disease progression and to reduce the risk of subsequent complications [21].

Lifestyle changes focused on improved diet quality can slow down or even halt (pre)diabetes progression [22,23,24]. For instance, an inverse association between plant-based diets (e.g., fresh vegetables, fruits) and T2DM evolution has been consistently highlighted [25,26,27,28,29]. Blueberries (BBs), one of the most popular berries due to their palatableness and nutritional/phytochemical composition, display a panoply of health-related properties in several metabolic diseases, including T2DM, as comprehensively reviewed elsewhere [30,31,32]. Briefly, their low caloric value pairs with an enriched content of dietary fibers and an array of polyphenolic secondary metabolites (e.g., flavonols, anthocyanins, phenolic acids) that greatly dictate BBs’ antioxidant and anti-inflammatory profiles [31,33,34,35]. Moreover, a wealth of experimental evidence openly points out the BBs-derived hypoglycemic and insulin-sensitizing effects [30,32,36,37]. However, the precise pathophysiologic mechanisms counteracted by BBs in early prediabetes progression are far from being disclosed. They are also consistently narrowed by the discrepancy in the experimental designs (e.g., disease stage, BBs doses and presentation forms, treatment duration) in both preclinical studies and clinical settings.

Recently, our group underscored that long-term BBs supplementation in a healthy condition triggered an expressive polyphenol-derived remodeling of hepatic mitochondrial bioenergetics [38]. Given the well-recognized hepatic mitochondrial impairments in overt T2DM, we hypothesized that BBs’ ability to counteract prediabetes would follow similar traits. This study aimed to shed light on cellular and molecular mechanisms paralleling the protective effects of BBs on glucose tolerance, insulin resistance, and lipid profile as early as prediabetes. To this end, we carried out an integrative preclinical approach encompassing functional and molecular readouts of gut health, hepatic metabolomics, and oxidative/inflammatory status, focusing on mitochondria, upon a clinically relevant BBs dose supplementation, in diet-induced prediabetes.

## 2. Materials and Methods

### 2.1. Blueberry Juice Preparation

Blueberries (*Vaccinium corymbosum* L., cultivar “Liberty”) were supplied from the same variety and in the same maturation stage by COAPE (Farming Cooperative of Mangualde, Mangualde, Portugal) and stored at −80 °C until use.

BBs juice (BJ) was obtained as described in a previous study [38]. Briefly, BBs were weighed, blended with sucrose solution (35%), and converted into juice to assure consumption of whole fruit parts (peel, pulp, and seeds). The volume of sucrose solution added was corrected every day to guarantee that 25 g/kg of BW of BBs were daily consumed by the animals. This BBs dose was selected based on a previous study of our group [38].

### 2.2. Phytochemical Screening of Blueberry Juice

The characterization of the phenolic compounds present in BJ was performed through HPLC-PAD-ESI/MS^n^ analysis, as previously described [38,39].

### 2.3. Animals and Experimental Design

Eight-week-old male Wistar rats (Charles River Laboratories, Barcelona, Spain) were pair-housed in ventilated cages, under controlled environmental conditions of temperature (22 ± 1 °C), humidity (50–60%), and light (12 h light-dark cycle) and *ad libitum* access to tap water and standard rodent chow. All animal procedures performed in this study were approved by the local (iCBR) Animal Welfare Body (ORBEA, #9/2018) and complied with the Animal Care National and European Directives and with the ARRIVE guidelines [40].

Following a one-week period of acclimatization, rats were arbitrarily assigned into 3 groups, in a 23-week protocol: control group, received standard chow and tap water (CTRL, *n* = 8); prediabetic group, received 35% sucrose (Hsu-84100; Sigma-Aldrich, St. Louis, MO, USA) in the drinking water plus standard chow until week 9, further supplemented by high-fat (HF) diet (58Y1, TestDiet, St. Louis, MO, USA) until week 23 (HSuHF, *n* = 10); and prediabetic group supplemented with BJ (HSuHF + BJ; *n* = 10), submitted to the same dietary regimen as the prediabetic group but received 25 g/kg BW/day of BJ (in 35% sucrose solution) between weeks 9 and 23. The development of prediabetes in rats, including the protocol of the dietary regimen and the duration, was performed based on our previous study [41].

Briefly, the high-fat diet contained 61.6% of energy as fat, 20.3% as carbohydrates (CH), and 18.1% as protein, with a total of 5.10 kcal/g (58Y1, TestDiet, St. Louis, MO, USA), whereas standard rat chow contained 8.6% of energy as fat and 67.9% as CH, 23.5% as protein, with a total of 3.15 kcal/g (4RF21 Mucedola, Milan, Italy).

Feed and beverage were provided *ad libitum* during the entire experimental protocol, except for the fasting periods. BW was weekly monitored; food and beverage consumption were recorded daily per cage throughout the protocol and used to calculate energy intake. The estimated values (calculated by dividing the total intake by two rats per cage) are presented per week.

### 2.4. Glucose Tolerance Test (GTT) and Insulin Tolerance Test (ITT)

At the first day of week 22, GTT was performed to assess the rats’ glucose tolerance. Rats submitted to a fasting period of 6 h were intraperitoneally (i.p.) injected with a solution of glucose (2 g/kg BW) and the blood glucose levels from the tail vein were recorded in samples taken immediately before (0 min) the injection and 30, 60, 90, and 120 min after, using an ACCU-CHEK^®^ Aviva portable glucometer (Roche Diagnostics, Mannheim, Germany). In addition, blood samples (≈30 μL) were collected before glucose challenge to assess fasting insulin levels.

At the first day of week 23, the in vivo peripheral insulin sensitivity was evaluated by ITT performed through the i.p. injection of insulin solution (0.75 units/kg BW; Actrapid Novo Nordisk, Bagsvaerd, Denmark) in 6-h fasted rats. Blood glucose levels were measured in the tail vein blood collected immediately before (0 min) the injection and 15, 30, 45, 60, and 120 min after, using the same glucometer, as previously described [41]. The area under the curve (AUC) of GTT (AUC_GTT_) and the rate constant for glucose clearance (KITT) were calculated as previously reported elsewhere [42,43].

### 2.5. In Vivo Hepatic Ultrasonographic Analysis

Rat liver ultrasound examination was performed using e General Electrics LOGIQe (GE Healthcare, Milwaukee, WI, USA), with a linear-array transducer with multifrequency (7.5–12 MHz). Before US examination, the rats were anesthetized by inhalation of 1.5% isoflurane by using an inhalation anesthesia system, kept under mild anesthesia during the data acquisition, and the abdomen was shaved by an electric shaver to reduce artifacts in the ultrasonography images. The liver and portal vein were analyzed in animals positioned in the supine position and the spleen in the right posterior oblique position, through multiple transversal and longitudinal scanning. A sound-conducting gel was applied, and the liver was assessed by placing the transducer just distal to the last right costal ribs and angling its beam cranially and obliquely, obtaining multiple transversal and longitudinal scans. A fundamental brightness mode (B-mode) was applied to all imaging. The acoustic focus was placed in the center of the liver and in the largest transverse cross-section of the spleen.

The ultrasound examination was performed by one blinded expert and the criteria for ultrasound diagnosis, including changes in the liver parenchymal echogenicity, focal steatosis, and diameter of the portal vein, were analyzed as previously described [44].

### 2.6. Biological Sample Collection

Serum samples were obtained by centrifugation (2300× *g*/15 min/4 °C) of whole blood samples (immediately collected after animal sacrifice by venipuncture from the jugular vein) and stored at −20 °C until assay. Liver and gastrointestinal tissues (duodenum and colon) were immediately excised, dissected into small pieces, and stored in conditions according to the assay’s requirements. The liver was firstly weighed and then divided into distinct pieces: one was immediately used for functional mitochondria assays; one was stored for NMR analysis; one was directly frozen in liquid nitrogen and stored at −80 °C until analysis for further protein and RNA extraction; one was kept in neutral buffered formalin solution (10%) to be used for histological analysis; and the remaining liver pieces were frozen by liquid nitrogen and stored at −80 °C. The liver weight relative to BW was calculated. During the last week of the experimental protocol, animals were put in metabolic cages to collect fecal samples, which were weighed and stored at −80 °C until processing.

### 2.7. Determination of Serum Metabolic Parameters

Serum samples were used to determine the postprandial glucose and triglycerides (TGs) by using automatic validated methods and equipment (Hitachi 717 analyzer, Roche Diagnostics GMBH, Mannheim, Germany), as previously described [10]. Serum insulin contents were quantified by using commercially available ELISA kits for rat samples (10-1250-01, Mercodia, Uppsala, Sweden).

### 2.8. Evaluation of Serum Redox Status

Total antioxidant status (TAS) in serum samples was estimated through the ferric reducing antioxidant potential (FRAP) assay, as previously described [45], with slight modifications. This method is based on the ability of the antioxidants contained in a sample to reduce ferric-tripyridyltriazine (Fe^3+^ -TPTZ) to a ferrous form (Fe^2+^) that develops an intense blue color, with maximum absorbance at 593 nm. In brief, the FRAP reagent was prepared freshly by the addition of acetate buffer (300 mM, pH 3.6), 10 mM of TPTZ (2.4.6-tripyridyl-s-triazine, T1253, Sigma-Aldrich, St. Louis, MO, USA) solution in 40 mM HCl and ferric chloride (FeCl_3_·6H_2_O, 236489, Sigma-Aldrich, St. Louis, MO, USA) solution (20 mM), at the ratio of 10:1:1 (*v*/*v*/*v*), respectively. In total, 10 μL of serum sample were added to 30 μL of distilled water and 300 μL of FRAP reagent in a 96-well microplate and incubated at 37 °C for 15 min; then, the absorbance was measured spectrophotometrically at 593 nm using a microplate reader (Synergy™ HT Multi-Detection Microplate Reader, BioTek^®^, Burlington, VT, USA). The antioxidant capacity of the samples was quantified from the calibration curve plotted using Trolox solution as the standard reference. Malondialdehyde (MDA) levels were performed through the thiobarbituric acid (TBA) reactive substances (TBARs) test. In total, 100 μL of serum were incubated (for 1 h) in a TBA solution, at room temperature (RT) and in dark conditions. The samples were then incubated at 90 °C for 60 min. Afterwards, the tubes were placed on ice for reaction cessation. In this assay, one MDA molecule chemically reacts with two TBA molecules, the final product being a molecule that can be spectrophotometrically quantified at 532 nm (pink pigment). The MDA concentration was calculated against a calibration curve using 1,1,3,3-tetramethoxypropane (108383, Sigma-Aldrich, St. Louis, MO, USA) as the external standard (range: 0.1–83.5 μM). The results were expressed as MDA/TAS ratio, a marker of oxidative stress [46].

### 2.9. ^1^H Nuclear Magnetic Resonance (NMR) Spectroscopy

Serum samples were prepared for the NMR analysis as described previously [41]. Liver metabolites were extracted from liquid N_2_ ground samples using a Folch extraction [47]. Polar extracts were lyophilized and dissolved in D2O phosphate buffer (0.2 M, pH = 7) supplemented with sodium fumarate (2 mM) used as internal standard. Samples for high-resolution ^1^H NMR analysis were loaded into 3 mm NMR-grade tubes.

1D-^1^H cpmg (Carr-Purcell-Meiboom-Gill spin-echo pulse sequence) NMR spectra were recorded by a 600 MHz (14.1 T) spectrometer (Agilent, Santa Clara, CA, USA) using a 3 mm indirect detection probe with a z-gradient. The spectra acquisition and processing were performed as previously described [38].

Recorded spectra were compared to the reference data from public databases, such as the Human Metabolome Database (HMDM), for spectral assignment [48]. In order to help spectral assignment, 2D homonuclear total correlation spectroscopy (TOCSY) spectra were also recorded [49]. Metabolites were identified according to Metabolomics Standards Initiative (MSI) guidelines for metabolite identification [50] and the identification levels are indicated in Supplementary Materials Tables S2 and S3.

Processed 1D cpmg spectra were bucketed using one-point bucket (0.6–9.0 ppm, with signal-free, water, and fumarate regions excluded) using Amix Viewer (version 3.9.15, Bruker Biospin GmbH, Rheinstetten, Germany) and aligned using the icoshift algorithm [51]. The resulting matrix was normalized by the total spectral area and also analyzed. Multivariate statistical analysis was applied on unit variance scaled matrix (SIMCA 14, Umetrics, Sartorius Stedim Biotech, Gottingen, Germany). Information on the global data structure was obtained from principal component analysis (PCA); in addition, partial least square discriminant analysis (PLS-DA) was used to evaluate class separation and identify the main metabolites involved in the discrimination of class. The qualitative measure of the predictive power (Q2) and the degree of fit to the data (R2) were given by a 7-fold internal cross-validation of the PLS-DA models, which were validated by the permutation test (*n* = 100) [52]. The corresponding PLS-DA loadings plots were obtained by multiplying the loading weight factors (w) by the standard deviation of the respective variables and were color-coded according to the variable importance in the projection (VIP). For quantitative assessment of metabolite variations between the groups, metabolite signals (VIP > 1) were integrated and normalized by the total spectral area. Outliers were excluded on the basis of the quality of the recorded NMR spectra according to the recommendations of MSI [50].

### 2.10. Extraction and Quantification of Gut Microbiota in Feces

#### 2.10.1. DNA Extraction from Stool

An NZY Tissue gDNA Isolation Kit (NZYtech, Lisbon, Portugal) was used to extract and purify genomic DNA from fecal samples, as previously reported [53]. DNA purity and quantification were evaluated with a NanoDrop spectrophotometer (Thermo Fisher Scientific, Wilmington, DE, USA).

#### 2.10.2. Real-Time PCR for Fecal Microbiota Analysis

Real-time PCR was performed by using a LightCycler FastStart DNA Master SYBR Green kit and a LightCycler instrument (Hoffman-La Roche Ltd., Basel, Switzerland) and the assay’s conditions were performed as previously described [53]. Primer sequences (Sigma-Aldrich, St. Louis, MO, USA) used to target the 16S rRNA gene of the bacteria and the conditions for PCR amplification reactions were previously described [38] and are listed in Table 1. Data are presented as the mean values of duplicate PCR analysis.

#### 2.10.3. Quantification of Fecal Short-Chain Fatty Acids (SCFAs) and Organic Acids 

SCFAs and organic acids (lactic and succinic acid) were evaluated using an Agilent 1200 series HPLC system with a refractive index—RI detector and with a UV detector, as previously described [53]. Quantification of fecal SCFAs was achieved by using calibration curves, and concentrations were expressed as mean micromoles/g of wet weight.

### 2.11. Evaluation of Intestinal Permeability Using FITC-Dextran

Intestinal integrity was determined by measuring the permeability of FITC-dextran. Rats from each group were fasted for 6 h and then gavaged with FICT-dextran solution (600 mg/kg, 4 kDa, Sigma-Aldrich), as previously described [54]. After a 4-h dosage, animals were anesthetized, and blood collected from the jugular vein. Blood samples were then centrifuged (2300× *g*, 10 min, 4 °C) to obtain serum. Aliquots of serum were diluted in PBS (1:1 *v*/*v*) and then added in a black 96-well microplate (Costar 96 back opaque). Subsequently, FITC-dextran was measured using a fluorescence spectrophotometer (BioTek Synergy HT, Winooski, VT, USA,) at an excitation and emission wavelength of 485 and 535 nm, respectively. Each sample was quantified in triplicate and the standard curve was obtained by diluting the serial concentrations of FTTC-dextran in non-treated serum diluted in PBS (1:1 *v*/*v*). The results are expressed as µg/mL.

### 2.12. Quantification of Serum Lipopolysaccharide (LPS) Concentration

Endotoxin LPS contents in serum samples were measured by using a Pyrochrome Lisate Mix, a quantitative chromogenic reagent, diluted in Glucashield^®^ buffer, as previously reported by our group [38].

### 2.13. Colon and Duodenum Analysis by Transmission Electron Microscopy (TEM)

Duodenum and colon samples collected after the sacrifice were immediately sectioned in small fragments of about 1 mm^3^ and fixed in 2.5% glutaraldehyde solution in 0.1 M phosphate buffer (pH = 7.2) for 2 h. Successive post-fixation was done in 1% osmium tetroxide (1.5 h) and in 1% aqueous uranyl acetate (1 h in the dark). After washing with distilled water, samples were dehydrated in a graded acetone series (30–100%) and embedded in an Epoxy resine (Fluka Analytical, Sigma-Aldrich, Darmstadt, Germany). Ultrathin sections obtained with a Leica EM UC6 (Leica Co, Vienna, Austria) ultramicrotome were then mounted on copper grids and stained with lead citrate 0.2% for 10 min. Observations were carried out on a TEM Tecnai G2 Spirit Bio Twin at 100 kV (FEI, Hillsboro, OR, USA), and images were processed using AnalySIS 3.2.

### 2.14. Immunohistochemical Staining

Cross-sections of rat colon (10 μm) were cut with a cryostat (Leica CM3050S, Nussloch, Germany) and fixed with an acetone:methanol mixture (1:1) at 20 °C for 2 min and then rehydrated in phosphate-buffered saline (PBS) (3 × 5 min). After rinsing, sections were permeabilized with 0.5% Triton X-100 in PBS for 15 min and blocked for 40 min with 4% non-fat milk in 20 mM Tris, pH 7.2, and 150 mM NaCl. Then, samples were incubated with primary antibodies: rabbit polyclonal anti-ZO-1 (ab96587, Abcam, Cambridge, MA, USA) and mouse monoclonal anti-occludin (OC-3F10, 33-1500, Life Tecnologies, Carlsbad, CA, USA) in PBS containing 1% BSA (4 °C overnight). After washing with PBS (3 × 5 min), the sections were incubated with the secondary fluorescent antibody Alexa Fluor 488-conjugated goat anti-rabbit IgG or Alexa Fluor 568-conjugated donkey anti-mouse IgG (1:200; Molecular Probes, Life Technologies, Paisley, UK) and 4′,6-diamidino-2-phenylindole (DAPI, nuclei dye, D1306, Invitrogen, Carlsbad, CA, USA), for 1 h at room temperature. Samples were then washed with PBS (3 × 5 min), and the slides mounted using the Glycergel mounting medium (Dako, C0563, Agilent, Santa Clara, CA, USA). Anti-ZO-1 and anti-occludin immunostaining samples were imaged using a confocal fluorescence microscope (LSM 710, Carl Zeiss, Gottingen, Germany).

### 2.15. Hepatic Histological Analysis

Hematoxylin-eosin (H&E) and Oil Red O staining were performed as previously described [41,55]. Sections were visualized with a Zeiss microscope Mod. Axioplan 2 (Zeiss, Jena, Germany).

### 2.16. Hepatic Triglycerides Quantification

Triglycerides contents in the liver samples were quantified by using a Triglycerides Colorimetric Assay kit (1155010, Cromatest^®^, Linear Chemicals, Barcelona, Spain), as described previously [56].

### 2.17. Hepatic SEM Analysis

Frozen liver sections were defrosted and mounted on an SEM stub using a double-sided carbon sticker. The specimens were analyzed using a compact variable-pressure scanning electron microscope (Hitachi, FlexSEM 1000, Tokyo, Japan) equipped with a cryostage at 15KV.

### 2.18. Hepatic Mitochondria Bioenergetics

Liver mitochondria were isolated in homogenization medium composed of 250 mM sucrose, 10 mM HEPES (pH 7.4), 0.5 mM EGTA, and 0.1% fat-free bovine serum albumin (BSA), as previously described [57,58]. EGTA and BSA were omitted from the final washing medium, adjusted at pH 7.4. After homogenization of the minced blood-free hepatic tissue, homogenates were centrifuged at 800× *g* for 10 min at 4 °C. Supernatant was collected and centrifuged at 10,000× *g* for 10 min at 4 °C to pellet mitochondria, which were resuspended in washing medium (250 mM sucrose and 10 mM HEPES, pH 7.4) and centrifuged again at 10,000× *g* for 10 min at 4 °C. Final pellet were resuspended in a final washing medium and immediately used. The integrity of mitochondrial (93 ± 2.5%) was assessed by quantifying citrate synthase (CS) activity, in the absence and presence of detergentof. The protein concentration was measured by using the biuret method calibrated with BSA [59].

#### 2.18.1. Mitochondrial Membrane Potential (ΔΨ)

The mitochondrial membrane potential (ΔΨ) was assessed using an ion-selective electrode to quantify the distribution of tetraphenylphosphonium (TPP^+^) as previously described [58,60] using an Ag/AgCl_2_ electrode as the reference. The parameters evaluated were the membrane potential (-mV), lag phase (seconds), repolarization (-mV), respiratory rates, and ΔΨ.

#### 2.18.2. Mitochondrial Respiration (Oxygen Consumption) and Permeability Transition (MPT)

Mitochondria oxygen consumption was polarographically monitored with a Clark oxygen electrode (Oxygraph, Hansatech Instruments Ltd., Cambridge, UK) as previously described [57]. Mitochondrial swelling was followed by changes in light scattering, as monitored spectrophotometrically at 540 nm, and the reaction conditions were performed as previously described [60]. All the experiments were performed in triplicate.

### 2.19. Gene Expression by Quantitative Real-Time PCR Analysis

Total RNA samples from liver and colon tissue were extracted using a PureLink RNA Mini Kit (12183018A, Ambion, Thermo Fisher Scientific, Carlsbad, CA, USA) as well as a Trizol protocol (93289, Sigma Aldrich; St. Louis, MO, USA), following the manufacturer’s instructions and as previously described [60].

### 2.20. Statistical Analysis

The distribution of continuous variables was analyzed using the Kolmogorov–Smirnov test to assess significant deviations from normality. Differences between experimental groups were compared using the nonparametric test Kruskal–Wallis test (followed by the Dunn’s test for multiple comparisons) for non-normally distributed data or the parametric test one-way ANOVA, followed by Bonferroni’s test for multiple comparisons) for normally distributed data. Repeated measures ANOVA, followed by the Bonferroni post-hoc test, was used to compare glucose levels throughout the GTT and ITT assays. The effect size was determined using Cohen’s d (*d*_Cohen_), which was calculated by dividing the mean difference between the groups by the pooled standard deviation [61]. The effect size was considered as small, medium, or large when *d*_Cohen_ was close to 0.2, 0.5, or 0.8, respectively. A large *d*_Cohen_ without a statistically significant *p* value means a higher variability and/or a small sample size. The G*Power software (version 1.3.9.4) was used to calculate the statistical power concerning fasting insulin levels. *p*-values ˂ 0.05 indicated statistical significance. Results are depicted as the mean ± standard error of the mean (SEM) and data analyses were performed using GraphPad Prism 6.0 software for Windows.

## 3. Results

### 3.1. Phenolic Composition of Blueberry Juice

The phenolic compounds were identified through HPLC-PDA-ESI/MS^n^. Results are displayed in the Supplementary Materials (Figure S1 and Table S1). The classes of phenolic compounds detected were hydroxycinnamic acids, flavonoids, and anthocyanins [38]. The identification of phytochemicals was based on UV and MS^n^ spectral data, complemented with the available literature. Chlorogenic acid (5-CQA) was the most abundant compound (peak 5) in BJ. Other caffeic and ferulic acid derivatives were also identified. The flavonoids present in BJ were essentially quercetin and myricetin O-glycosides. The anthocyanins present in BJ showed UV spectra and fragmentation patterns characteristic of delphinidin, malvidin, and petunidin derivatives.

### 3.2. Effects of BJ on Body Weight and Caloric Intake in HSuHF-Fed Rats

During the experimental protocol, the body weight (BW) of the animals was monitored weekly. Before feeding the HF diet and BJ being provided, similar BW values were recorded among groups. Afterwards, the increase in BW over time was more pronounced in both groups fed with the HF diet (HSuHF and HSuHF + BJ) than in the CTRL group. As shown in Table 2, the delta BW (final minus initial) was significantly higher in the HSuHF group compared to the CTRL group (*p* < 0.05). However, BJ supplementation had no significant effect on BW gain.

HSuHF showed a higher total caloric intake as compared to the CTRL group (*p* < 0.0001). Prediabetic animals supplemented with BJ ingested an equivalent total amount of calories compared to the HSuHF group, which therefore supports the similar BW between these two experimental groups (Table 2). Significantly lower feed intake was observed in animals of HSuHF when compared to the CTRL group (*p* < 0.0001). Meanwhile, HSuHF + BJ animals ingested more pellets of HF diet than those fed with the HSuHF diet alone (*p* < 0.001) (Table 2). Regarding cumulative drink intake, higher liquid ingestion was found in the HSuHF group compared to the CTRL group (*p* < 0.0001) and, on the other hand, HSuHF + BJ decreased beverage intake, when compared to the HsuHF animals (*p* < 0.001). Concerning the macronutrient composition, the HsuHF rats displayed a significantly higher intake of carbohydrates (*p* < 0.0001) and lipids along with a lower protein (*p* < 0.0001) intake when compared to the CTRL group. Owing to increased HF diet ingestion, the HSuHF + BJ groups consumed significantly more proteins (*p* < 0.001) and lipids, together with a slight reduction in carbohydrates contents (reflected by the lower BJ ingestion) when compared to the HSuHF group (Table 2).

### 3.3. Effects of BJ on the Glycemic and Insulinemic Profile in HSuHF-Fed Rats

**Figure 1:**
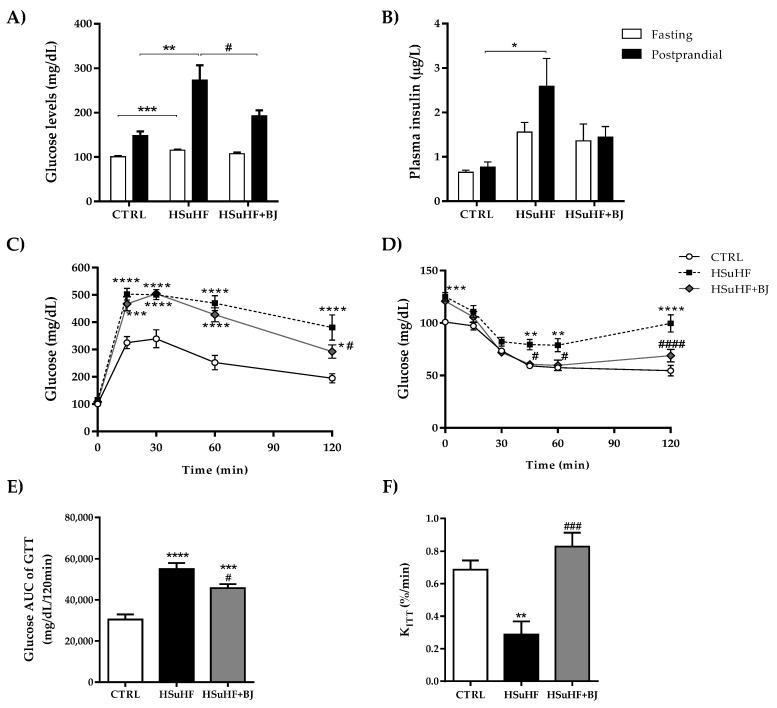
Effects of BJ supplementation on the glycemic and insulinemic profile in HSuHF-fed rats. Fasting and postprandial glucose levels (**A**). Fasting and postprandial insulin levels (**B**). Blood glucose levels throughout the glucose tolerance test (GTT) (**C**); Blood glucose levels throughout the insulin tolerance test (ITT) performed through intraperitoneal injection of insulin (0.75 U/kg body weight) (**D**); area under the curve (AUC) of the blood glucose level during GTT, calculated between baseline and 120 min after glucose bolus (2 mg/kg) (**E**); kITT representing the glucose clearance rate (%min) (**F**) in CTRL, HSuHF, and HSuHF + BJ groups. Results are expressed as the mean ± SEM. (*n* = 8–10/group; in fasting insulin levels: *n* = 3–4/group were used); * *p* < 0.05, ** *p* < 0.01, *** *p* < 0.001, and **** *p* < 0.0001 vs. CTRL; # *p* < 0.05, ### *p* < 0.001, and #### *p* < 0.0001 vs. HSuHF. CTRL: control. HSuHF: high sucrose / high-fat. BJ: blueberry juice.

Fasting and postprandial blood glucose concentrations were significantly higher in the HSuHF group when compared to the CTRL rats (*p* < 0.001 and *p* < 0.01, respectively). However, although there was no statistical difference on fasting glycemia, BJ supplementation had a positive effect on fed glucose levels, being significantly lowered (*p* < 0.05) when compared to the HSuHF group (Figure 1A). Regarding serum insulin, there was a trend to increased fasting levels in the HSuHF group, although it did not reach statistical significance (Figure 1B), eventually due to the small sample size used for this parameter (statistical power value of 0.644). Still, in the fed state, an increase in insulin values was observed in the HSuHF group when compared to the CTRL rats (*p* < 0.05), an effect that was ameliorated in the HSuHF + BJ group. To further determine the impact of BJ supplementation on glucose tolerance and insulin sensitivity, we next performed the glucose and insulin tolerance tests.

As expected, HSuHF displayed glucose intolerance and insulin resistance as revealed by the higher glucose concentrations throughout the 2 h of GTT and ITT (Figure 1C,D, respectively) and further supported by both the significantly higher area under the curve (AUC) of GTT (*p* < 0.001 vs. CTRL group) (Figure 1E) and lower glucose disappearance rate (kiTT), a measure of insulin sensitivity (*p* < 0.01 vs. CTRL group) (Figure 1F). Otherwise, no statistical changes were found in the fasting glucose and insulin levels between the two experimental groups. However, in the HSuHF + BJ-treated rats, glucose intolerance and insulin sensitivity were alleviated when compared to the HSuHF group, as reflected by both the lower AUC of GTT (*p* < 0.05) and significantly higher kITT values (*p* < 0.001) (Figure 1C–F). Collectively, these findings indicate that BJ supplementation had significant protection against glucose intolerance and insulin resistance in the prediabetic rats.

### 3.4. Effects of BJ on the Serum Lipid Profile and Redox Status Markers in HSuHF-Fed Rats

**Figure 2:**
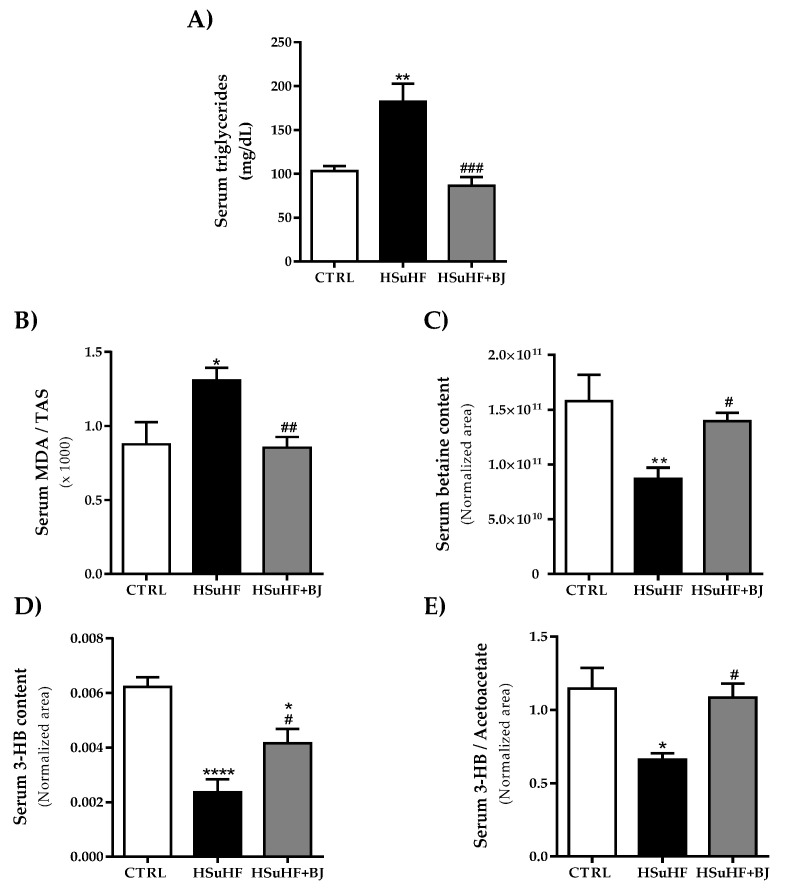
Effects of BJ on serum triglycerides contents (**A**) and on serum redox marker (**B**). Serum contents of betaine (**C**). Serum levels of 3-hydroxybutyrate (3-HB) (**D**) and 3-HB/acetoacetate ratio (**E**) in CTRL, HSuHF, and HSuHF + BJ groups. Results are expressed as the mean ± SEM. (*n* = 8–10/group); * *p* < 0.05, ** *p* < 0.01 and **** *p* < 0.0001 vs. CTRL; # *p* < 0.05, ## *p* < 0.01, and ### *p* < 0.001 vs. HSuHF. CTRL: control. HSuHF: high sucrose / high-fat. BJ: blueberry juice.

Regarding serum TGs, significantly higher concentrations were found in the prediabetic group when compared to the CTRL group (*p* < 0.05). The HSuHF-induced hypertriglyceridemia was reverted with BJ supplementation for 14 weeks, with significantly lower postprandial TGs values *versus* those found in HSuHF-fed animals (*p* < 0.01) and were similar to those observed in the CTRL group (Figure 2A).

Serum total antioxidant status (TAS) and MDA levels were used to assess the total antioxidant capacity and lipid peroxidation. We determined the serum MDA/TAS ratio as a redox status marker. The HsuHF group presented significantly higher values of the serum MDA/TAS ratio (*p* < 0.05 vs. CTRL group). This effect was restored by BJ supplementation in the HSuHF + BJ-treated group, which showed a significantly lower MDA/TAS ratio (*p* < 0.01) compared to the HSuHF-fed animals, suggesting a restored antioxidant profile (Figure 2B).

Moreover, we further performed a nontargeted proton nuclear magnetic resonance spectroscopy (^1^H NMR)-based metabolomic approach in serum samples to identify possible endogenous metabolite alterations and to define putative molecular mechanisms underpinning BJ protection. The identified serum metabolites as well as their chemical shifts are listed in the Supplementary Materials (Table S2). To identify the key metabolite changes between the groups, a partial least squares-discriminate analysis (PLS-DA) was performed. Accordingly with the multivariate data analysis, the most pronounced findings were related to betaine and ketone bodies levels. There was a significant reduction in betaine levels in serum samples from HSuHF-fed animals compared to the CTRL group (*p* < 0.05), which was slightly recovered in serum from HSuHF + BJ-treated animals (*p* < 0.05 vs. HSuHF group). The levels of 3-hydroxybutyrate (3-HB) and the 3-HB/acetoacetate ratio were significantly lowered in the serum of the HSuHF-fed group compared to the CTRL group. Conversely, a remarkable recovery in the contents of these metabolites was found in the serum of HSuHF + BJ -treated animals when compared to the prediabetic group (*p* < 0.05 vs. HSuHF group), reaching similar values from those found in the control group. Moreover, the relative concentrations of valine, histidine, lactate, and N-acetylglycoproteins were significantly lower in the HSuHF group, when compared with control animals; however, BJ supplementation had no significant impact on their contents in serum, as can be seen in the Supplementary Materials Table S2.

### 3.5. Effects of BJ on Gut Microbiota Composition and Intestinal Integrity in HSuHF-Fed Rats

We assessed the composition of the bacterial community by real-time PCR and the SCFAs and organic acids by HPLC in fecal samples that were collected at the end of the experimental protocol. As shown in Table 3, the GM composition of HSuHF-fed rats was modestly altered compared to the control´s GM. The hypercaloric diet-fed animals presented a trend of a decreased relative abundance of Firmicutes and *Bacteroidetes* phylum independently of the supplementation with BJ, albeit no significant differences were found when compared to the CTRL group. Moreover, a trend was observed towards an increase in the Firmicutes/*Bacteroidetes* ratio and *Enterococcus* in HSuHF-fed rats but without statistical significance when compared to the control animals (*p* > 0.05).

The abundance of *Akkermansia* was significantly lower in HSuHF-fed animals (*p* < 0.05) compared to the control animals. Nonetheless, in spite of the BJ addition showing a trend to counteract the loss of the proportions of *Akkermansia* induced by hypercaloric feeding, a modest antimicrobial effect of BJ was encountered in the fecal GM composition from BJ-treated animals. Interestingly, the gut microbiota of the HSuHF + BJ group was less abundant in *Bifidobacterium* and *Prevotella* when compared to the gut microbiota of untreated prediabetic animals (Table 3).

Despite these subtle changes observed in the bacterial community from HSuHF groups, the SCFA profile revealed a lowered fecal content of butyric and propionic acids when compared to the CTRL group (*p* < 0.01 and *p* < 0.05, respectively). Moreover, the BJ supplementation was unable to alter the fecal SCFAs contents compared to the HSuHF-untreated rats (Table 3); however, the addition of BJ resulted in a significant decrease in the fecal contents of succinic acid when compared to the gut microbiota of the untreated HSuHF group (*p* < 0.05), likely due to the loss of the succinate-producing *Prevotella* ssp.

**Figure 3:**
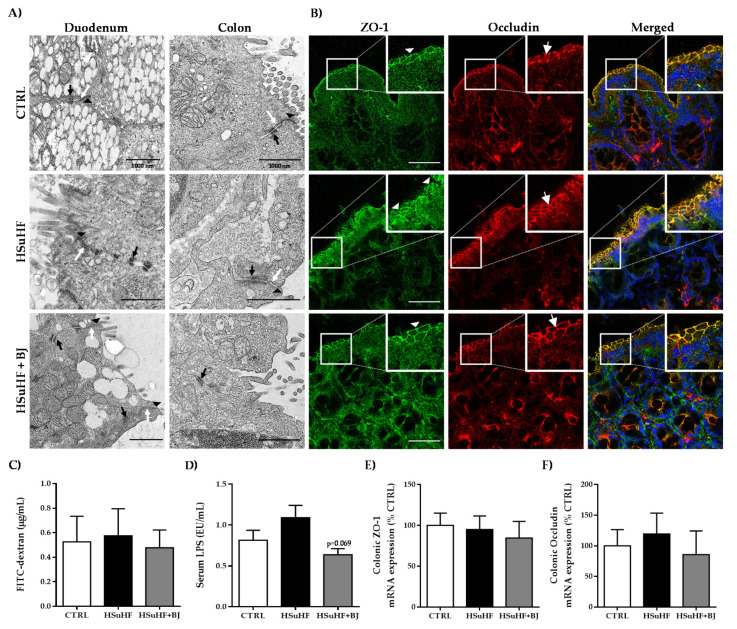
Impact of BJ on gut barrier structure and intestinal permeability. Representative TEM images of ultrastructural analysis of tight junctions (black arrowhead), adherens junctions (white arrow), and desmosomes (black arrow) in duodenum and colon sections (**A**) (scale bar: 1000 nm). Representative confocal images of immunohistochemistry staining of ZO-1 (white arrowhead) and occludin (white arrow) in colon sections (**B**) (scale bar: 50 µm). Serum FITC-dextran concentrations (**C**), serum LPS levels (**D**), colonic mRNA expression of ZO-1 (**E**), and occludin (**F**) in CTRL, HSuHF, and HSuHF + BJ groups. Results are expressed as the mean ± SEM. (*n* = 8–10/group); *p* = 0.069 vs. HSuHF group. CTRL: control. HSuHF: high sucrose/high-fat. BJ: blueberry juice.

The junctional complexes, namely tight junctions, adherens junctions, and desmosomes, were visualized in the duodenum and colon sections through transmission electron microscopy (TEM) and representative images of the ultrastructure of the duodenum and colon are shown in Figure 3A. Iintact junctional complexes were found in all epithelial cells observed in each experimental group. The data of the epithelial barrier integrity were further supported by the data of immunofluorescence staining of the tight junction proteins ZO-1 and occludin.

At the epithelial cell’s periphery, an identical distribution of ZO-1 and occludin was found between the groups (Figure 3B), which was also validated by the unchanged mRNA expression of colonic tight junctions, ZO-1, and occludin between the control and the experimental groups (Figure 3E,F, respectively). We also evaluated the intestinal permeability by measuring the paracellular passage of the 4 kDa-dextran labeled with FITC to serum and by quantifying the serum LPS contents, which is a membrane component found in Gram-negative bacteria. There were no changes in the serum FITC-dextran concentrations 4 h after oral FITC dextran administration between the groups, suggesting the absence of intestinal paracellular permeability changes induced by the hypercaloric diet and by BJ (Figure 3C). These results were further corroborated by the unchanged serum LPS levels; despite a trend to increased serum LPS contents in the HSuHF group *versus* the CTRL, the differences found did not reach statistical significance. However, a trend of decreased LPS levels in animals supplemented with BJ was observed (*p* = 0.069 vs. HSuHF group) (Figure 3D).

### 3.6. Effects of BJ on Hepatic Lipid Steatosis in HSuHF-Fed Rats

**Figure 4:**
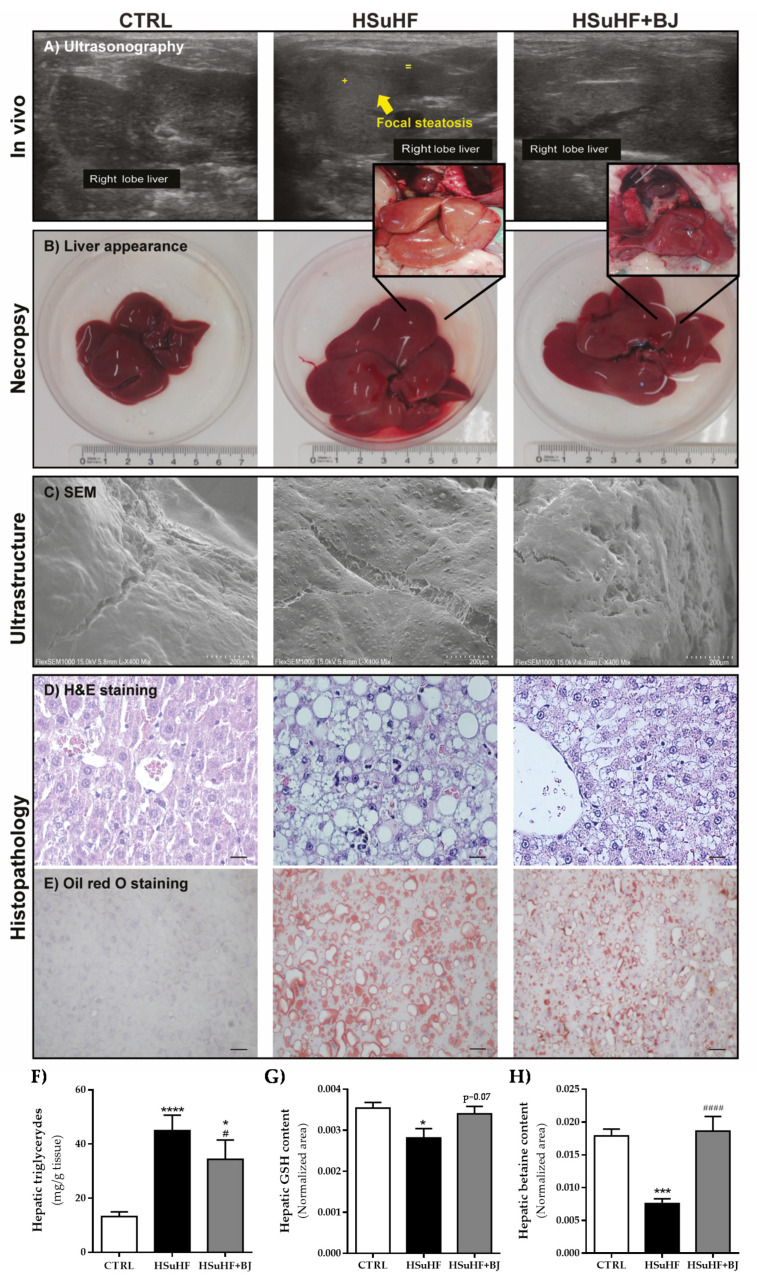
Impact of BJ on liver appearance and hepatic findings. Representative photographs of in vivo ultrasonography (**A**), macroscopic liver appearance during dissection and tissue collection (**B**), SEM images of rat liver tissue (**C**), and Hematoxylin–eosin and (**D**) Oil Red O staining (**E**) of liver sections from representative rats from each group (×400 magnification; scale bar = 20 µm); hepatic triglycerides contents (**F**); hepatic GSH (**G**) and betaine contents (**H**) per group. Results are expressed as the mean ± SEM. * *p* < 0.05, *** *p* < 0.00,1, and **** *p* < 0.0001 vs. CTRL group; # *p* < 0.05 and #### *p* < 0.0001 and *p* = 0.07 vs. HSuHF group. CTRL: control. HSuHF: high sucrose/high-fat. BJ: blueberry juice.

The relative liver weight (normalized to BW) was unchanged between HSuHF and CTRL (28.84 mg/Kg ± 1.31 and 25.75 mg/Kg ± 0.57, respectively); however, in the HSuHF + BJ-treated rats, there was a reduced liver/BW value (24.24 mg/Kg ± 0.72) when compared to the prediabetic animals (*p* < 0.01). Ultrasound imaging, macroscopic appearance, ultrastructure analysis, and histological examination by H&E staining and Oil red O staining were performed to analyze hepatic changes between experimental groups. As shown in Figure 4A, through ultrasound imaging, we found that the HSuHF groups displayed signs of focal steatosis, which was almost absent in the HSuHF + BJ-fed animals. Moreover, during dissection and tissue collection, macroscopic changes were notorious in the liver of the HSuHF group, namely related to the color, which became slightly yellow, along with abnormal enlargement and a distinct texture when compared with CTRL and HSuHF + BJ animals’ livers. This latter was also validated through the SEM technique, where ultrastructural changes were found in the livers of HSuHF animals (Figure 4C). As expected, while CTRL animals had normal livers, the HSuHF group showed hepatic fat vacuoles with inflammatory cell infiltration and enhanced lipid accumulation, as observed in H&E and Oil red O-stained slides. BJ supplementation improved the macroscopical appearance and markedly reduced the ballooning and lipid deposition in the hepatocytes of the HSuHF + BJ group (Figure 4D,E). Furthermore, these results were also supported by the assessment of hepatic TGs contents. In agreement with serum samples, we observed that the hypercaloric diet increased liver TGs levels (*p* < 0.0001 in HSuHF-fed animals vs. CTRL groups), while BJ supplementation resulted in significantly lowered hepatic TGs values (*p* <0.001) when compared to the HSuHF group, despite remaining higher than the CTRL group (*p* < 0.05) (Figure 4F). Nevertheless, these findings demonstrated that BJ relieved lipid accumulation in hypercaloric diet-induced prediabetic rats.

Multivariate analysis was performed on ^1^H NMR data for hepatic metabolic profile comparison between the CTRL and experimental groups. Metabolomic analysis of the liver samples demonstrated a reduced hepatic glutathione level in HSuHF rats compared to the CTRL group. Although missing statistical significance, there was a clear trend of increased hepatic GSH levels in the HSuHF + BJ rats (*p* = 0.07 vs. HSuHF-fed rats) (Figure 4G). In addition, the relative levels of betaine were significantly decreased in the liver of prediabetic animals, which were totally recovered in HSuHF + BJ-treated rats (Figure 4H).

### 3.7. Impact of BJ on Hepatic Mitochondrial Function in HSuHF Rats

**Figure 5:**
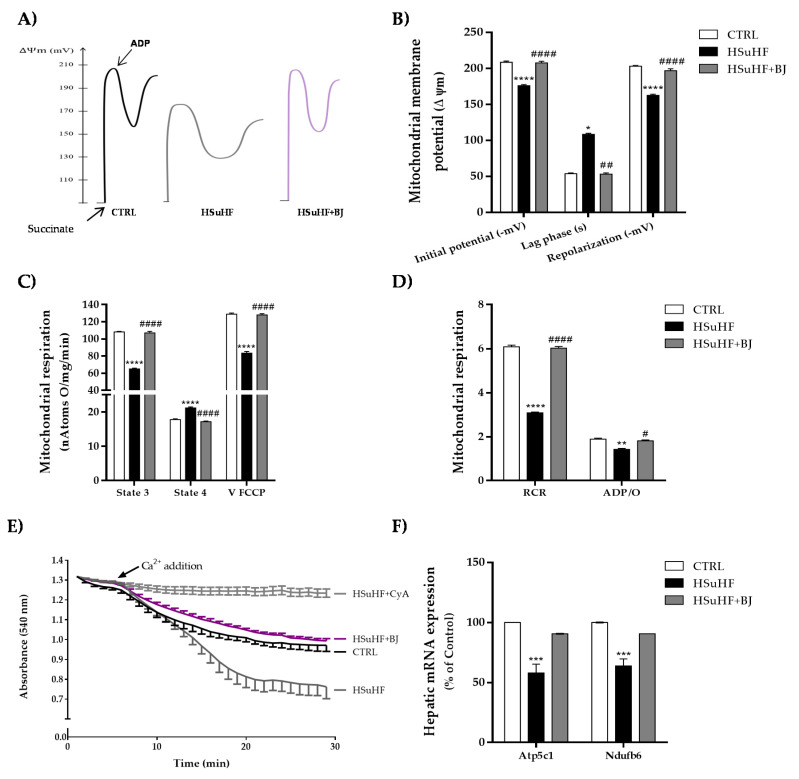
Effects of BJ on mitochondrial function parameters, including mitochondrial membrane potentials and lag phase (**A**,**B),** mitochondrial respiration parameters (**C**,**D**), and susceptibility to the induction of mitochondrial permeability transition (MPT) (**E**); hepatic mRNA expression of genes involved in the mitochondrial electron transport chain (**F**). Results are expressed as the mean ± SEM. (*n* = 6–8/group). * *p* < 0.05, ** *p* < 0.01, *** *p* < 0.001, and **** *p* < 0.0001 vs. CTRL group; # *p* < 0.05, ## *p* <0.01, and #### *p* < 0.0001 vs. HSuHF group. CTRL: control. HSuHF: high sucrose/high-fat. BJ: blueberry juice. FCCP: carbonylcyanide-P-trifluoromethoxyphenylhydrazone. RCR: respiratory control ratio. ADP/O: adenosine diphosphate to oxygen ratio.

To elucidate whether BJ consumption induced protective effects on hepatic mitochondrial function, we assessed several bioenergetic parameters in isolated hepatic mitochondria. Oxidative phosphorylation relies on the generation of a transmembrane electrochemical potential (ΔΨ, in the form of a proton gradient), associated with molecular oxygen consumption. Compared to the control group, the hepatic mitochondria from HSuHF groups showed a significant reduction in mitochondrial initial ΔΨ (after substrate addition) and ΔΨ after repolarization (mitochondrial capacity to establish ΔΨ after ADP phosphorylation). These parameters were clearly reverted by BJ supplementation in the HSuHF + BJ-treated rats. In addition, a significant increase in the lag phase time (which represents the time required for ADP phosphorylation) was noticed in hepatic mitochondria from HSuHF-fed animals compared to the CTRL groups (*p* < 0.05), suggesting a compromised hepatic mitochondrial function in HSuHF-fed animals. In contrast, mitochondria from HSuHF + BJ-treated animals showed a shorter lag-phase, which indicated faster phosphorylation activity (Figure 5A,B).

The putative alterations in the respiratory chains and oxidative phosphorylative system of mitochondria isolated from the control and both experimental groups were also determined by following the oxygen consumption in the presence of succinate. Mitochondria isolated from HSuHF-fed rats displayed a significant decline in respiratory state 3 (ADP-stimulated respiration) and FCCP-uncoupled respiratory rates together with an increase in respiratory state 4 (in the ADP absence, corresponding to the resting state) (*p* < 0.0001), when compared to mitochondria from the control group (Figure 5C). Consequently, the respiratory control ratio (RCR) was significantly lowered (*p* < 0.0001) in mitochondria isolated from the livers of HSuHF-fed animals (Figure 5D). Additionally, the oxidative phosphorylation efficiency estimated by the ADP/O ratio was significantly decreased in mitochondria from HSuHF-fed rats as compared to control animals (*p* < 0.01) (Figure 5D), reflecting an uncoupling between respiration and ADP phosphorylation. BJ supplementation effectively reversed the effects of the HSuHF diet on mitochondrial respiration parameters. When compared to rats fed simply with a hypercaloric diet, the results showed that mitochondria from HSuHF + BJ-treated rats had a significant rise in respiratory state 3, FCCP-uncoupled respiration, RCR, and the ADP/O ratio, as well as reduced state 4 respiration (Figure 5C,D).

Figure 5E illustrates the induction of mitochondrial swelling as a marker for permeability transition pore induction. The isolated hepatic mitochondria from HSuHF in the presence of 20 nmol Ca^2+^ and 5 mM succinate underwent a pronounced decrease in light scattering, reflecting the ability to further induce the opening of the mitochondrial membrane permeability transition pore. The hypercaloric diet-induced mitochondrial swelling was completely prevented by BJ supplementation, as detected by an increase in light scattering. Furthermore, hypercaloric diet-induced swelling was sensitive to cyclosporin A (Figure 5E), reflecting calcium-dependent MPT induction.

Collectively, these findings prompted us to evaluate at the transcriptional level of genes regulating the mitochondrial energetics in the liver. We, therefore, analyzed the expression of genes encoding for subunits of the mitochondrial respiratory chain complexes by RT-PCR. The expression of Ndufb6 and ATp5c1, which encode proteins of complex I (subunit NADH:ubiquinone oxidoreductase) and complex V (mitochondrial ATP synthase), respectively, was significantly downregulated (*p* < 0.001) in the liver tissue from HSuHF-fed rats when compared to the CTRL animals. As shown in Figure 5F, even though it failed to reach statistical significance, in HSuHF + BJ-treated rats, there was a trend of increased expression of both mitochondrial phosphorylation-related genes, compared to the HSuHF-untreated group (Ndufb6 *d*_Cohen_ =2.65 and Atp5c1 *d*_Cohen_ =2.54).

### 3.8. Effects of BJ on Hepatic mRNA Expression of Inflammation-Related Genes

**Figure 6:**
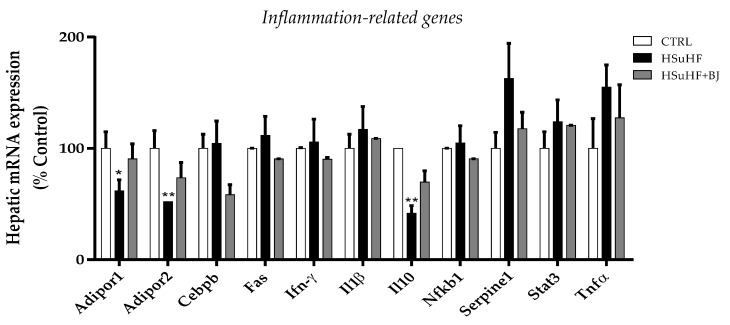
Hepatic mRNA expression of genes related to the inflammatory response of the CTRL, HSuHF, and HSuHF + BJ groups. Data are presented as the mean ± SEM. (*n* = 6–8/group). * *p* < 0.05, ** *p* < 0.01 vs. CTRL group. CTRL: control. HSuHF: high sucrose/high-fat. BJ: blueberry juice.

We assessed the impact of BJ supplementation on the hepatic mRNA expression of genes related to the inflammatory response. The mRNA expression of adiponectin receptors (Adipor1 and Adipor2) and the anti-inflammatory gene interleukin-10 (IL-10) was significantly repressed in the liver of HSuHF-fed rats (*p* < 0.05 and *p* < 0.01) when compared to the CTRL group. Surprisingly, despite a trend to recover the mRNA expression of these genes, no statistical changes were detected in the rats of the HSuHF + BJ group when compared to the HSuHF ones (Adipor1 *d*_Cohen_ = 0.99; Adipor2 *d*_Cohen_ = 0.60; IL-10 *d*_Cohen_ = 1.43). Similarly, the mRNA expression of Cebpb, which is an important transcription factor that regulates the expression of genes involved in the immune and inflammatory response, was not altered in HSuHF-fed rats (*p* > 0.05 vs. CTRL group); however, this tended to decrease in the liver of HSuHF + BJ-treated rats (Cebp *d*_Cohen_ = 1.51). Furthermore, there was a slight but not significant increase in the mRNA levels of plasminogen activator inhibitor 1 precursor (Serpine1) and tumor necrosis factor-α (Tnf-α) in the HSuHF rats compared to the animals of the CTRL and HSuHF + BJ groups, despite being restored upon BJ treatment (Serpine *d*_Cohen_ = 0.94; Tnf-α *d*_Cohen_ = 0.15). The expression of the tumor necrosis factor receptor superfamily member 6 precursor Tnfrsf6 (Fas), Ifn-γ, interleukin-1 beta (Il-1β), Nf-kB, and Stat3 mRNA was unaffected by either the hypercaloric diet or BJ treatment (Figure 6).

### 3.9. Effects of BJ on Hepatic mRNA Expression of Glucose Metabolism and Insulin Signaling-Related Genes

**Figure 7:**
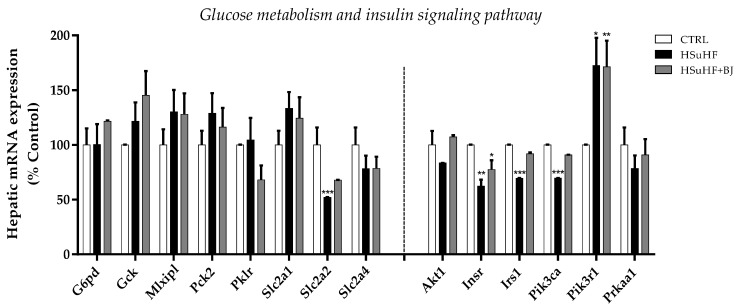
Hepatic mRNA expression of genes related with glucose metabolism and the insulin signaling pathway of CTRL, HSuHF, and HSuHF + BJ groups. Data are presented as mean ± SEM (*n* = 6–8/group). * *p* < 0.05, ** *p* < 0.01, and *** *p* < 0.001 vs. CTRL group. CTRL: control. HSuHF: high sucrose / high-fat. BJ: blueberry juice.

As glucose homeostasis and insulin sensitivity were compromised in prediabetic animals and improved upon BJ supplementation, the relative mRNA expression of genes involved in glucose metabolism as well as in the insulin-signaling pathway was determined (Figure 7). Concerning genes encoding rate-limiting glycolytic enzymes, the mRNA levels of glucokinase (Gck) and pyruvate kinase L/R (Pklr) were not significantly different between the three groups. In addition, no significant differences in the mRNA expression of the rate-limiting gluconeogenic enzyme, the mitochondrial isoform of phosphoenolpyruvate carboxylase 2 (Pck2), as well as in the mRNA of Mlxipl (gene encoding carbohydrate response element-binding protein—ChREBP) were identified in the liver of the experimental groups. Among the genes required for glucose uptake, the expression of Slc2a2 (gene encoding GLUT2) was remarkably decreased in HSuHF-fed rats compared to the CTRL group (*p* < 0.001). There was a slight increase of the Slc2a2 mRNA expression in the livers of HSuHF + BJ-treated animals compared to the HSuHF-fed rats (Slc2a2 *d*_Cohen_ = 84.48), although this was still lowered when compared to the CTRL group. Conversely, a slight increase in the hepatic mRNA expression of Slc2a1 (encoding glucose transporter GLUT1) was found in HSuHF-fed rats (*p* = 0.066 vs. CTRL group); however, it was unaltered upon BJ intake. Despite a trend of decreased mRNA expression of Slc2a4 (gene encoding GLUT4) when animals were fed with the hypercaloric diet, statistical significance was not detected between the experimental groups (Figure 7).

The expression of genes involved in insulin signaling, including Insr (insulin receptor), IRS-1 (insulin receptor substrate-1), and Pik3ca (catalytic subunit alpha isoform of phosphoinositide-3-kinase—PI3K), was notably declined in prediabetic animals (*p* < 0.01 and *p* < 0.001 vs. CTRL group, respectively). BJ provided to the HSuHF-fed animals partly reversed this reduction when compared to the untreated prediabetic animals (Insr *d*_Cohen_ = 0.63; Irs1 *d*_Cohen_ = 9.89; Pi3kca *d*_Cohen_ = 125.59). A similar tendency was found in the mRNA expression of Akt1 (protein kinase B—isoform 1) and Prkaa1 (protein kinase AMP-activated catalytic subunit alpha 1); however, no statistical differences were detected between the experimental groups. The mRNA expression of Pik3r1 (gene encoding the p85 alpha regulatory subunit of PI3K) was significantly enhanced in HSuHF-fed animals (*p* < 0.05 vs. CTRL group), which was not restored upon BJ treatment (Figure 7).

### 3.10. Effects of BJ on the Hepatic mRNA Expression of Lipid Metabolism-Associated Genes

**Figure 8:**
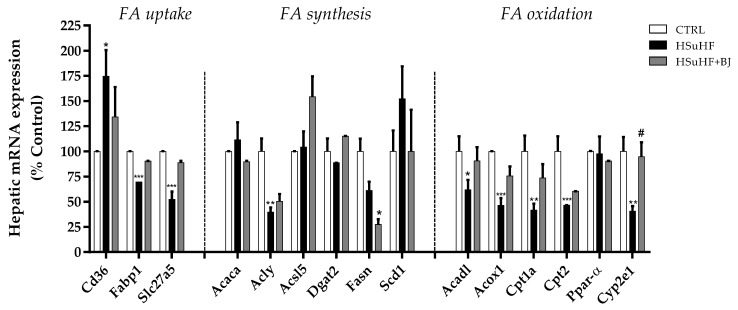
Hepatic mRNA expression of genes related with lipid metabolism of the CTRL, HSuHF, and HSuHF + BJ groups. Data are presented as the mean ± SEM. (*n* = 6–8/group). * *p* < 0.05, ** *p* < 0.01, and *** *p* < 0.001 vs. CTRL group; # *p* <0.05 vs. HSuHF group. CTRL: control. HSuHF: high sucrose/high-fat. BJ: blueberry juice.

To explore the molecular basis for the lipid-lowering effect hepatoprotection of BJ, the mRNA expression levels of several lipid metabolism-related genes, including those involved in hepatic fatty acids (FAs) uptake, synthesis, and β-oxidation, were determined by RT-PCR in the liver tissue (Figure 8). The mRNA expression of fatty acid translocase Cd36, which mediates FA uptake across the cell membrane, was significantly enhanced in the HSuHF-fed rats (*p* < 0.05 HSuHF vs. CTRL group), whereas the mRNA expression of hepatic fatty acid-binding protein 1 (Fabp1), and fatty acid transport protein 5 (Slc27a5) was markedly lowered in HSuHF-fed rats (*p* < 0.001 vs. CTRL). There were no statistical differences in the hepatic mRNA expression of Cd36, FATP5, and Slc27a5 in the HSuHF + BJ-treated group when compared with the HSuHF-fed one, despite a trend of suppression of these hypercaloric diet-induced mRNA changes of FATP5 (*d*_Cohen_ = 92.19) and Slc27a5 (*d*_Cohen_ = 2.50).

Regarding the genes involved in the fatty acid synthesis, the mRNA expression of Acly, which encodes ATP-citrate synthase that catalyzes the synthesis of cytosolic acetyl-CoA from citrate, was significantly decreased in HSuHF-fed rats compared to the CTRL ones (*p* < 0.01) and remained lower when animals were treated with BJ. In contrast, in the HSuHF group, there was a trend to increased mRNA expression of Scd1, which encodes the stearoyl-coenzyme A desaturase 1, which catalyzes a rate-limiting step in the biosynthesis of monounsaturated fats. This increase tended to be abolished when HSuHF-fed animals were supplemented with BJ since the mRNA levels reached similar values to those of the CTRL group; however, no statistical significance was achieved when compared to the prediabetic group (Scd1 *d*_Cohen_ = 0.68). The expression of other genes involved in lipogenesis (Acaca, Acsl5, and Dgat2) remained unchanged either by feeding with the hypercaloric diet or BJ supplementation. The mRNA expression of fatty acid synthase (Fasn) was significantly decreased in HSuHF + BJ-treated rats compared to the CTRL group (*p* < 0.05), with no significant changes in HSuHF rats. Regarding lipogenic transcription regulator, the expression of SREBF1 (encoding sterol regulatory element-binding protein-1c, SREBP-1c) remained unaltered in the hepatic tissue of animals from the HSuHF and HSuHF + BJ groups (Figure 8).

Compared with the CTRL group, HSuHF-fed animals showed a significant decrease in the hepatic expression of fatty acid oxidation-related genes, including long-chain acyl coenzyme A dehydrogenase (Acadl), acyl-CoA oxidase (Acox-1), carnitine palmitoyltransferase 1 (Cpt1), and carnitine palmitoyltransferase 2 (Cpt2) (*p* < 0.05 vs. CTRL group). Conversely, despite not reaching statistical differences, the HSuHF + BJ-treated rats showed a trend of normalizing the mRNA expression of these key enzymes when compared to the HSuHF-fed rats (Acadl *d*_Cohen_ = 0.99; Acox-1 *d*_Cohen_ = 1.39; Cpt1 *d*_Cohen_ = 1.29 and Cpt2 *d*_Cohen_ = 84.20). Surprisingly, the mRNA expression of PPARα (peroxisome proliferator-activated receptor-alpha), a nuclear receptor regulator of the fatty acid oxidation pathway in the liver, was unchanged between the experimental groups (Figure 8).

## 4. Discussion

In the present study, we dissected the putative mechanisms underpinning BJ protection in a prediabetic rat model, focusing on the gut microbiota composition and intestinal barrier integrity as well as on hepatic structural and functional data, with a particular emphasis on mitochondrial bioenergetics.

**Figure 9:**
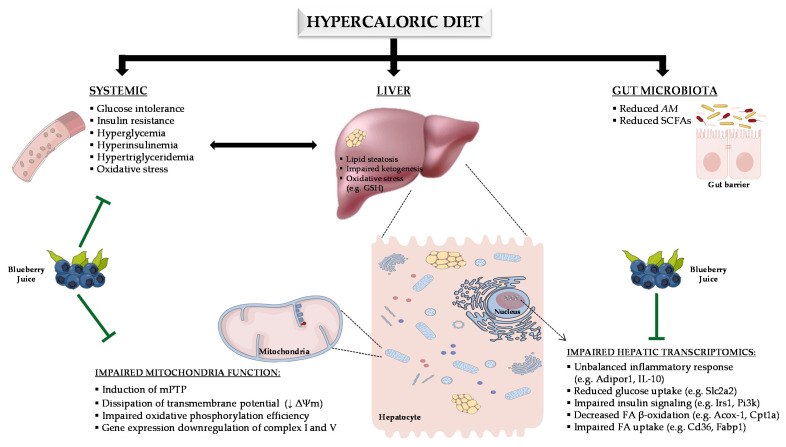
Blueberry counteracts the hypercaloric diet-evoked metabolic dysregulation by improving the glycemic, insulinemic, and lipid profile along with reduced hepatic steatosis. Moreover, together with restoring systemic and hepatic antioxidant metabolites, BBs positively modulate the gene expression of key targets of inflammation, insulin signaling, and fatty acid metabolism as well as rescuing the mitochondria function in the liver. Abbreviations: *AM*, *Akkermansia muciniphila*; FA, fatty acids; SCFAs, short-chain fatty acids; mPTP, mitochondrial permeability transition pore. _┴_ protective/inhibitory effects induced by BJ.

As expected, the hypercaloric diet induced an increase in BW gain in the prediabetic rats, as a result of a higher intake of calories, especially carbohydrates and lipids, when compared with the control animals. These changes were not prevented in the HSuHF rats under BJ supplementation, which presented unchanged BW and calorie consumption. Previous studies showed contradictory data regarding the impact of different sources of BBs on BW gain in animal models, with some of them reporting reductions [62,63,64] and others no significant effect [65,66,67]. In our study, the use of a liquid form (juice) of BBs consumption may explain the compensatory increase of food intake and the lack of an impact on animals’ BW. In fact, it has been shown that fluid calories have lower satiating power and that fibers are lost during fruit-to-juice processing [68,69,70], which collectively may explain the reduced protective effect of BBs against excess intake and weight gain. In this study, we also replicated the major metabolic features of rat models of diet-induced prediabetes [41,71]. In fact, the HSuHF-fed rats presented postprandial hyperglycemia, glucose intolerance (as viewed by the GTT), postprandial hyperinsulinemia, and reduced insulin sensitivity (as suggested by the ITT), as well as hypertriglyceridemia (Figure 9). The addition of BJ to the hypercaloric diet-fed prediabetic animals promoted a reduction of fed glucose levels, ameliorated glucose intolerance, and enhanced insulin sensitivity. These findings agree with previous studies in animal models of obesity and T2DM [67,72,73,74].

In addition, our results are consistent with those showing that BBs prevent postprandial TGs accumulation in the serum in rats fed hypercaloric diets [75,76,77]. The phytochemical composition of BBs, enriched in phenolic compounds (namely anthocyanins, among others) and fibers, is likely to explain the beneficial metabolic effects of BBs [78,79,80] due to their antioxidant and prebiotic properties being very well documented [32,81,82,83,84]. However, the precise mechanisms underlying the protection of BBs against deregulation of glucose, insulin, and lipids, especially in the prediabetic state, remain to be elucidated. In the present study, we chose to deepen some of the most promising hypotheses, starting from the potential to modulate the gut microbiota and related mechanisms.

Changes in the composition or diversity/richness of gut microbiota (called dysbiosis) have been associated with several extraintestinal disorders, including metabolic diseases, such as obesity and diabetes [85]. Diet is a major modulator of GM, and hypercaloric diets evoke dysbiosis [86,87]; on the contrary, fiber and polyphenols—which are abundant in blueberry—are promoters of symbiosis [88,89].

Regarding the prediabetic rats, we did not obtain all the typical markers of dysbiosis described in other studies using both animal models and human samples [5,6,7,90]. For example, the HSuHF-treated rats presented an unchanged relative abundance of Firmicutes to Bacteroidetes. Notwithstanding, consistent with our data, a similar unchanged Firmicutes:Bacteroidetes ratio was observed in Wistar rats fed with HF or HSuHF diets for 10 weeks [91]. In addition, ultrastructural analysis of the gut by TEM revealed maintenance of the epithelial barrier integrity. The intestinal barrier’s function is based on an intact epithelial lining, which is comprised of epithelial cells that are joined at different levels of the intercellular junction by cell-to-cell structural adhesions, such as tight junctions, adherens junctions, and desmosomes [92]. TJs are dynamic and highly organized complexes, consisting of various proteins, including ZO-1 and occludin, which allow selective transport across the intestinal barrier. Moreover, the mRNA expression levels of ZO-1 and occludin and their distribution at the periphery of epithelial cells were unchanged. In line with these results, the measure of endotoxemia and the FITC-dextran permeability assay showed that the intestinal barrier was functionally still intact. These results could be eventually explained by the early disease stage, although some studies have already described dysbiosis in prediabetic states [5,90,93]. Even though, there was a reduced abundance of *Akkermansia muciniphila* (AM) in the HSuHF-treated animals, a bacterial species that has been negatively correlated with glucose tolerance, insulin resistance, and obesity [94,95,96]. Furthermore, there was a reduced fecal content of SCFAs (Figure 9), which is in line with what has been described in states of prediabetes and diabetes [6,90,97,98]. Regarding the HSuHF animals treated with BJ, the prebiotic properties previously described in other conditions [62,99,100,101,102] were not replicated in our study. In fact, the reduced abundance of AM found in the HSuHF-treated animals was not prevented, which was accompanied by a significant reduction of *Prevotella* spp. and *Bifidobacterium.* These distinct bacterial communities are typically associated with SCFAs production, which were not recovered by BJ supplementation in the HSuHF rats. In addition, fecal succinate was reduced in the BJ-fed animals; since *Prevotella* spp. belongs to a group of succinate-producing bacteria [103], the reduction in fecal succinate in BJ-treated prediabetic animals can be eventually derived from the suppressed *Prevotella* abundance. In addition, there was a reduced overall count of bacteria (universal), which might be eventually explained by the antimicrobial properties described for some polyphenols that are abundant in BBs, such as quercetin and hesperidin [104,105,106]. Although it is important in the future to study models with other characteristics, the results of our study, using this early disease model, show that gut microbiota modulation cannot explain the metabolic improvement observed in prediabetic animals when treated with BJ. In this sense, we moved forward to check the expected antioxidant properties of BJ and find possible crosslinks with other mechanisms that regulate the metabolism of glucose and lipids.

Hyperglycemia and hypertriglyceridemia are key drivers of glucotoxicity and lipotoxicity, which are among the main causes of oxidative stress and inflammation, two mechanisms closely linked with the (pre)diabetic state [107,108]. In our model, the serum MDA/TAS ratio was higher in HSuHF rats and ameliorated in BJ-treated animals (Figure 9). In addition, we found a depletion of hepatic GSH levels in prediabetic rats, an effect that was prevented by BJ supplementation (HSuHF + BJ group). Another important finding of this study from NMR analysis was the increment of taurine in the hepatic tissue of BJ-treated animals. This endogenous metabolite has diverse cytoprotective actions, such as antioxidant activity linked to improved mitochondrial function (e.g., decreases the superoxide generation by mitochondria) [109,110,111]. Furthermore, we also found a decreased betaine content in the serum and liver of HSuHF rats, an effect that was prevented in BJ-treated animals. Betaine is a methyl donor that can be obtained directly from the diet or by choline oxidation [112,113]. This metabolite plays a role in the hepatic remethylation of the methionine-homocysteine cycle by betaine-homocysteine methyltransferase (BHMT), producing carnitine, a key factor in fatty acid metabolism related to the transfer of long-chain fatty acids into mitochondria for subsequent β-oxidation [114]. In line with our results, lower levels of hepatic betaine were observed in Zucker rats [115] and in HFD-induced obese mice [116,117]. Indeed, it has been shown that betaine supplementation improves hepatic steatosis and insulin resistance both in in vivo [117,118,119,120] and in vitro studies [117]. Betaine levels were restored in BJ-treated animals, which could also be explained by the enriched amount of betaine present in BBs [113]. In addition, 3-hydroxibutyric acid (3-HB), a ketone body that represents the main alternative energy substrate to glucose and is a by-product of hepatic FA oxidation, was recovered in the rats of the HSuHF + BJ group. In agreement with previous studies [121,122], the reduced serum levels of 3-HB in HSuHF rats might suggest impaired FA metabolism and a degree of hepatic mitochondrial dysfunction since ketone bodies are produced from acetyl-Coa via ketogenesis, which mainly occurs in the hepatic mitochondria [8]. The recovery of 3-HB levels and the 3-HB/acetoacetate ratio in the rats of the HSuHF group when supplemented with BJ suggests an improvement in FA oxidation and mitochondrial function. Thus, our findings agree with the expected antioxidant properties of BBs and further points to hepatic mechanisms regulating the metabolism, including at the mitochondrial level.

Several studies have indicated a close relationship between mitochondrial dysfunction or defects in mitochondrial biogenesis and the pathogenesis of insulin resistance and associated metabolic disorders [16,123]. It has been consistently considered that the health-promoting effects of some polyphenols extend to mitochondria and may represent a valuable and attractive nutritional strategy to prevent or hinder the metabolic impairments underpinning mitochondrial dysfunction [124,125]. Indeed, some polyphenols can modulate several mitochondrial processes, including mitochondrial biogenesis, oxidative phosphorylation, mitochondrial membrane potential (ΔΨ), and ATP synthesis, among others [126,127,128]. Our group previously showed that a long-term intake of BBs phytochemicals triggered a hepatic mitochondrial-related metabolic transcriptomic reprogramming in healthy rats [38]. In light of these considerations, we then focused our attention on the BJ effects on the hepatic tissue towards functional and molecular readouts from a mitochondria perspective in a prediabetic setting.

Our results revealed that the prediabetic animals displayed hepatic steatosis, viewed mainly by an increased hepatic content of TGs, which was paralleled by an impaired mitochondrial function and insulin resistance (Figure 9). Noticeably, BJ supplementation for 14 weeks alleviated hepatic steatosis, which is in accordance with other studies [75,76]. The hepatic lipid overload represents a key driver for lipotoxicity, which together with glucotoxicity are closely associated with mitochondrial dysfunction [129]. Furthermore, hypercaloric diet-induced prediabetic animals display an impairment in hepatic mitochondrial bioenergetics machinery, which contributes to a redox imbalance and fosters ectopic lipid storage [123,130]. Accordingly, we showed that the hypercaloric diet elicited considerable impairments in mitochondrial functional activity comprising disturbances in mitochondrial electric potentials and respiration, associated with decreased state 3 and VFCCP, which may result from dysfunction in the electron transport chain complexes or due to an uncoupling of oxidative phosphorylation, as suggested by the decreased RCR and ADP/O. Moreover, the hypercaloric diet-induced changes in respiratory parameters rendered mitochondria more susceptible to Ca^2+^-induced mitochondrial permeability transition. mPTP opening, resulting from unbalanced cytoplasmic ion levels, mainly Ca^2+^, potentiates membrane potential dissipation and uncoupled oxidative phosphorylation, resulting in ATP depletion. Although discrepancies have been reported in other studies [131,132,133,134], several pieces of evidence are in line with our data showing impaired HF-induced bioenergetics [123,135,136].

BJ supplementation restored these detrimental effects observed in the prediabetic animals, as viewed by: (i) recovery of the transmembrane electrochemical potential, indicating protection of passive permeability to protons (proton leakage); (ii) increased oxidative phosphorylation efficiency (restored state 4 and increased RCR and ADP/O; and (iii) restored activity of the electron transport chain (increase state 3 and VFCCP). The protection of mitochondrial function exerted by BJ is also sustained by the reduced mPTP opening, protecting mitochondrial from hypercaloric diet-induced swelling. This effect can be mediated by antioxidant protection against oxidative damage in the mitochondria membrane [127]. Consistent with the mitochondrial dysfunction, prediabetic animals showed significantly decreased mRNA levels of NDufb6 (a subunit of complex I) and ATP5C1 (a subunit of complex V), which were recovered upon BJ supplementation, indicating that BJ improved hepatic mitochondrial integrity and function (Figure 9).

To gain further insight into the metabolic protection prompted by BJ, we also evaluated the expression of genes involved in glucose metabolism and insulin signaling. Significantly reduced hepatic expression of genes involved in insulin signaling (e.g., Ins, Irs1, Pi3ca) was observed in the HSuHF group, an effect that was partly restored in the liver of BJ-treated animals. Similar results were found in the liver, pancreas, and white adipose tissue of HF diet-fed mice after blueberry-leaf extract intake [137]. Furthermore, we showed that BJ was able to normalize the hepatic mRNA expression levels of Glut2, the main glucose transporter in the liver that was downregulated in prediabetic rats. This effect may account for the normalization of hepatic glucose levels by promoting insulin-stimulated glucose uptake and by restoring insulin sensitivity.

Besides being the cell’s powerhouse, mitochondria are also pivotal for oxidative catabolism of amino acids, ketone bodies synthesis, and FA breakdown through β-oxidation [8,129,138]. With more efficient mitochondria, hepatocytes can handle more nutrients (namely lipids) and consequently avoid their accumulation. The reduced deposition of hepatic TGs along with high levels of ketone bodies in BJ-treated prediabetic animals pave the way to an efficient shift towards fatty acid β-oxidation and ketone synthesis (Figure 9). These effects are in line with the recovery of serum 3-HB levels and the 3-HB/acetoacetate ratio in the rats of the HSuHF + BJ group. To complement these observations, we analyzed the liver expression of a panel of genes related to lipid metabolism, including FA uptake, synthesis, and oxidation. Previous studies have shown that fatty acid translocase/CD36 overexpression increases hepatic TGs storage by increasing FA uptake [139], which is in line with our results showing increased mRNA CD36 expression in prediabetic animals. In the livers of BJ-treated animals, a trend toward normal values was obtained, which was probably due to decreased systemic FA. Conversely, the normalization of the mRNA expression of fatty acid-binding protein-1 (Fabp1) and fatty acid transporter 5 (FATP5/Slc27a5) in BJ-treated animals suggests a compensatory hepatocyte FA uptake, preventing cytotoxicity promoted by binding of long-chain fatty acids. While most evidence shows the BBs phytochemicals can protect from steatosis by suppressing the expression of genes involved in lipogenesis [137,140,141,142,143], in this study, we found unchanged mRNA expression of key genes involved in lipogenesis, such as Fasn, Acaca, ACSL5, and SREBP-1C, together with unaltered mRNA expression of Dgat2 and Scd1. Thus, considering the above data of FA uptake and synthesis, our results suggested that the lipid-lowering activity of BJ cannot be explained by the ability to suppress fatty acid synthesis, contrasting with the effects of BJ on FA β-oxidation. In fact, the HSuHF rats presented decreased mRNA expression of peroxisomal Acox1, as well as mitochondrial ACADL, CPT-1, and CPT-2, reflecting an imbalance from the substrate influx exceeding fat burning, thus favoring the ectopic lipid accumulation and storage in hepatocytes. BJ supplementation was able to rescue the mRNA expression of some of these genes, which collectively may contribute to the improvement of hepatic FA β-oxidation in prediabetic rats (Figure 9). These results are consistent with other studies showing beneficial lipid homeostasis of BBs polyphenols or anthocyanins extracts in mice models of metabolic diseases [62,76,140,141,142,144]. Moreover, the prevention of serum and hepatic lipid accumulation in the BJ-treated animals of our study was accompanied by changes in some genes towards the improvement of the liver inflammatory profile, suggesting a protective effect of BJ against lipotoxicity. Indeed, the reduced lipid loading in the BJ-treated group could eventually explain the improved performance of hepatocytes, namely the slight changes in gene expression data.

Future studies are warranted to elucidate the open questions and to overcome the specific limitations of this study. Microbial analysis should be performed using metagenomic sequencing technology to provide stronger information about the functional diversity of the bacterial community. In addition, further work should dissect the possibility that the decreased consumption of 35% sucrose in the BJ group might be partially responsible for the improved metabolic phenotype. In addition, alternative players possibly involved in the impact of the consumption of sugar-sweetened beverages should be explored. For instance, it would be interesting to assess the effects of BJ on the activity of ChREBP and the expression of other key regulators of hepatic carbohydrates metabolism, such as PEPCK1 and G6PC.

## 5. Conclusions

In the current study, using a hypercaloric diet-induced rat model, we found that long-term BJ supplementation at 25 g/day for 14 weeks afforded protective effects against prediabetes by: (i) improving glucose homeostasis and insulin sensitivity, (ii) preventing hypertriglyceridemia and liver lipid deposition, and (iii) promoting strong antioxidant effects on serum and hepatic levels. The beneficial metabolic effects of BJ in this early disease stage could not be attributed to a correction of gut dysbiosis or prevention of leaky gut. Instead, the improved hepatic mitochondrial function, accompanied by restored FA oxidation and ketogenesis, may contribute to explaining the reduction of hepatic steatosis and hyperlipidemia, as well as the amelioration of insulin resistance and glucose metabolism. Our data provide evidence that amelioration of hepatic mitochondrial function could be a crucial mechanism by which BJ exerts antidiabetic properties in early stages (prediabetes). The present work paves the way to support the use of BBs to counteract prediabetes progression.

## Figures and Tables

**Table 1:** Primer sequences and real-time PCR conditions used for gut microbiota analysis.

Bacterial Group	Primer Sequence (5′-3′)	PCR Product Size (bp)	AT (°C)
Firmicutes	ATG TGG TTT AAT TCG AAG CA	126	45
	AGC TGA CGA CAA CCA TGC AC		
*Bacteroidetes*	CAT GTG GTT TAA TTC GAT GAT	126	45
	AGC TGA CGA CAA CCA TGC AG		
*Clostridium*	GCA CAA GCA GTG GAG T	239	45
	CTT CCT CCG TTT TGT CAA		
Universal	AAA CTC AAA KGA ATT GAC GG	180	45
	CTC ACR RCA CGA GCT GAC		
*Enterococcus*	CCC TTA TTG TTA GTT GCC GCC ATC ATT	144	50
	ACTCGT TGT ACT TCC CT TGT		
*Prevotella*	CAC RGT AAA CGA TGG ATG CC	513	50
	GGT CGG GTT GCA GAC C		
*Bifidobacterium*	CGC GTC YGG TGT GAA AG	244	50
	CCC CAC ATC CAG CAT CCA		
*Roseburia*	TAC TGC ATT GGA AAC TGT CG	230	50
	CGG CAC CGA AGA GCA AT		
*Lactobacillus*	GAG GCA GCA GTA GGG AAT CTT C	126	55
	GGC CAG TTA CTA CCT CTA TCC TTC TTC		
*Akkermansia*	CAG CAC GTG AAG GTG GGG AC	327	55
	CCT TGC GGT TGG CTT CAG AT		

AT, annealing temperature; bp, base pairs; PCR, polymerase chain reaction.

**Table 2:** Body weight, cumulative total calories, food, drink, and nutrient intakes of the animals after 23 weeks of the experimental period.

Parameters	CTRL	HSuHF	HSuHF + BJ
**Body weight (BW)**			
Initial (g)	285.40 ± 19.80	287.30 ± 14.71	286.20 ± 16.68
Final (g)	510.00 ± 20.90	602.00 ± 32.89	589.40 ± 31.75
Delta BW (g)	224.60 ± 10.67	314.60 ± 23.62 *	303.20 ± 17.85 *
**Cumulative intakes**			
Total calories (Kcal/rat/week)	515.50 ± 5.55	715.20 ± 10.12 ****	742.00 ± 16.03 ****
Food (g/rat/week)	163.00 ± 1.77	60.44 ± 1.63 ****	76.42 ± 1.94 ****^, ###^
Drink (mL/rat/week)	214.10 ± 4.30	328.10 ± 7.51 ****	285.70 ± 12.98 ****^, ###^
**Macronutrients cumulative intakes**			
Carbohydrates (Kcal/rat/week)	350.20 ± 3.93	573.90 ± 14.01 ****	548.40 ± 21.73 ****
Lipids (Kcal/rat/week)	44.19 ± 0.47	98.73 ± 8.76	132.40 ± 12.35
Proteins (Kcal/rat/week)	121.10 ± 1.30	51.92 ± 1.61 ****	65.84 ± 1.90 ****^, ####^

Results are expressed as mean ± SEM. (*n* = 8–10/group); * *p* < 0.05 and **** *p* < 0.0001 vs. CTRL; ^###^ *p* < 0.001 and ^####^ *p* < 0.0001 vs. HSuHF. CTRL: control. HSuHF: high sucrose/high-fat. BJ: blueberry juice.

**Table 3:** Gut bacterial microbiota groups and SCFA contents in feces of CTRL, HSuHF, and HSu + BJ rats.

Parameters	CTRL	HSuHF	HSuHF + BJ
**Bacteria groups (Log10 copies /ng of DNA)**			
Universal	6.188 ± 0.317	5.941 ± 0.178	5.140 ± 0.190 *^, #^
Firmicutes	7.316 ± 0.175	7.043 ± 0.123	6.785 ± 0.201
*Bacteroidetes*	4.641 ± 0.183	4.167 ± 0.262	4.142 ± 0.185
Firmicutes/Bacteroidetes	1.605 ± 0.042	1.778 ± 0.086	1.643 ± 0.033
*Bifidobacterium*	1.706 ± 0.119	2.53 ± 0.02	1.280 ± 0.156 ^##^
*Prevotella*	2.736 ± 0.286	2.817 ± 0.286	1.820 ± 0.225 ^#^
*Lactobacillus*	4.087 ± 0.795	4.507 ± 0.350	4.317 ± 0.169
*Akkermansia*	4.823 ± 0.271	3.561 ± 0.373 *	3.859 ± 0.141
*Clostridium leptum*	4.817 ± 0.176	4.714 ± 0.195	4.604 ± 0.138
*Roseburia*	3.628 ± 0.277	3.829 ± 0.306	3.901 ± 0.322
*Enterococcus*	2.620 ± 0.494	3.096 ± 0. 240	2.496 ± 0.230
**SCFAs (mg/g feces)**			
Acetic acid	1.24 ± 0.11	0.97 ± 0.11	0.68 ± 0.07 **
Butyric acid	0.07 ± 0.01	0.02 ± 0.01 **	0.02 ± 0.00 **
Propionic acid	0.03 ± 0.00	0.01 ± 0.00 *	0.01 ± 0.00 *
Succinic acid	7.01 ± 0.79	8.86 ± 1.33	4.82 ± 0.76 ^#^

Results are expressed as mean ± SEM. (*n* = 8–10/group); * *p* < 0.05, ** *p* < 0.01 vs. CTRL; ^#^ *p* < 0.05, ^##^ *p* < 0.01 vs. HSuHF. CTRL: control. HSuHF: high sucrose / high-fat. BJ: blueberry juice. SCFA: short-chain fatty acids.

## Data Availability

The data present in this current study are available from the corresponding author upon reasonable request.

## Supplementary Materials

The following are available online at https://www.mdpi.com/article/10.3390/nu13124192/s1, Figure S1: Chromatographic profile of phenolic compounds in BJ, obtained with HPLC-PDA-ESI/MS^n^ (UV, 320 nm); Table S1: Polyphenol compounds identified in Blueberry juice by HPLC-PDA-ESI/MS^n^; Table S2: Metabolites detected in serum samples by ^1^H-NMR analysis; Table S3. Metabolites detected in liver samples by ^1^H-NMR analysis. References [145,146,147,148,149] are cited in the supplementary materials
