# Supplementary material for: Blueberry Counteracts Prediabetes in a Hypercaloric Diet-Induced Rat Model and Rescues Hepatic Mitochondrial Bioenergetics
Source: Nutrients. 2021 Nov 23;13(12):4192. doi: 10.3390/nu13124192 (PMC8706913; doi:10.3390/nu13124192)
Supplement: Supplementary file 1 [file nutrients-13-04192-s001.zip › nutrients-1409909-supplementary.pdf]

Supplementary Materials

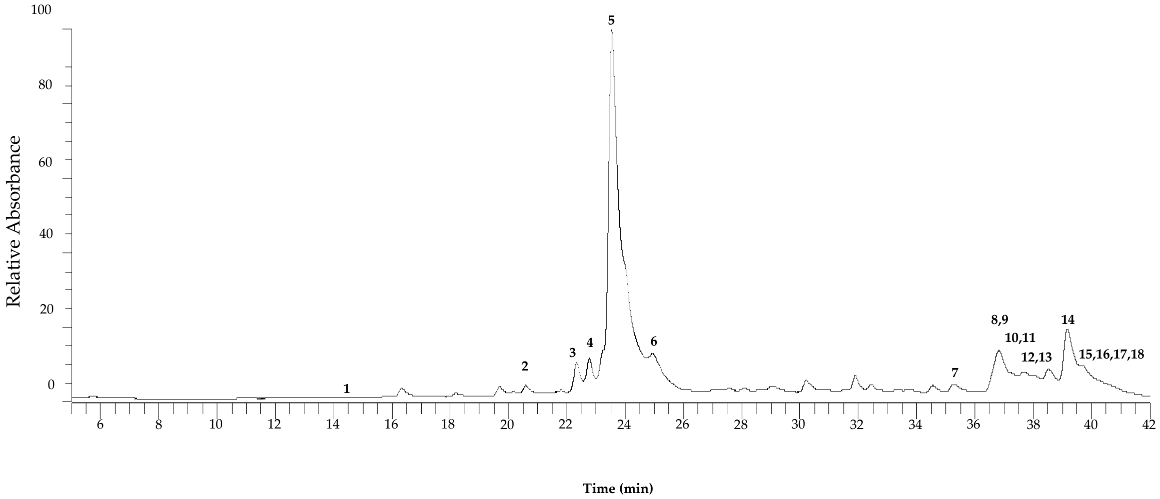

Figure S1. Chromatographic profile of phenolic compounds in BJ, obtained with HPLC-PDA-ESI/MSn (UV, 320nm).

Table S1. Polyphenol compounds identified in blueberry juice by HPLC-PDA-ESI/MS^n.

Peak	R_t (min)	λ_{max} (nm)	ESI-MSn[a] [*m/z* (relative abundance, %)]			Attempt to identify	Refs.
			Precursor Ion [M-H]$^-$	MS^2	MS^3		
1	14.22	248sh, 271, 280sh	315	153 (100)	123 (100)	Protocatechuic acid hexoside	[145]
2	20.59	250, 280, 299sh, 322sh	341	179 (100)	135 (100)	Caffeic acid hexoside	[145]
3	22.33	246, 285, 309, 320sh	341	179 (100)	135 (100)	Caffeic acid hexoside	[145]
4	23.23	248, 291, 299, 326	355	217 (55), 193 (100), 175 (20)	178 (22), 149 (50), 134 (100)	Ferulic acid hexoside	[146]
5	23.54	245, 291sh, 303sh, 326	353	191 (100)	173 (100), 171 (24)	5-CQA	[35]
6	24.93	249, 291, 321	353	191 (100)	173 (87), 171 (17), 155 (14), 127 (100), 111 (29), 109 (34), 93 (49), 87 (13), 85 (64)	*cis*-5-CQA	[35]
7	35.27	218, 249, 384sh, 347	463	317 (78), 316 (100)	287 (38), 272 (13), 271 (100), 270 (40), 179 (36), 151 (11)	Myricetin -O-deoxyhexoside	[147]
8	36.21	218, 250, 278sh, 335	463	337 (16), 318 (10), 317 (65), 316 (100), 315 (13), 301 (18), 300 (27)	288 (12), 287 (25), 271 (100), 270 (25)	Myricetin -O-deoxyhexoside	[147]
9	36.84	220, 253, 267sh, 287sh, 354	463	302 (12), 301 (100), 300 (21)	273 (17), 257 (16), 179 (100), 151 (60)	Quercetin-O-hexoside	[148]
10	37.33	219, 249, 267, 282sh, 353, 527	---	---	---	Delfinidin derivative	[149]
11	37.72	220, 248, 270, 281sh, 348, 529	569	523 (68), 491 (100)	343 (12), 331 (17), 329 (100), 328 (22)	Malvidin hexoside	[149]
12	38.50	219, 251, 267, 281sh, 352, 527	433	301 (100)	273 (13), 257 (10), 179 (100), 151 (71)	Quercetin-O-pentoside	[148]
13	38.67	219, 251, 267, 281sh, 352, 527	---	---	---	Delfinidin – pentoside	[149]
14	39.16	220, 254, 264sh, 284sh, 350, 530	447	302 (11), 301 (100), 300 (19)	283 (10), 273 (17), 179 (100), 151 (65)	Quercetin-O-deoxyhexoside	[148]
15	39.30	220, 254, 264sh, 284sh, 350, 530	---	---	---	Delfinidin	[149]
16	39.69	219, 253, 266sh, 283sh, 350, 530	447	302 (15), 301 (100), 300 (19)	283 (15), 273 (20), 257 (12), 229 (10), 179 (100), 151 (74)	Quercetin-O-deoxyhexoside	[148]
17	39.90	219, 253, 266sh, 283sh, 350, 530	---	---	---	Petunidin	[149]
18	39.90	219, 253, 266sh, 283sh, 350, 530	---	---	---	Malvidin	[149]

Identification based on the UV-Vis spectra, the molecular weight, and the fragmentation patterns, which are according to references (Refs).

Table S2. Metabolites detected in the serum samples by ^{1}H-NMR analysis.

Serum Metabolites	Chemical shift (ppm)	CTRL	HSuHF	HSuHF + BJ
**Amino acids and BCAA metabolism**				
Isoleucine	0.997 - 1.001(d)	4.550e-5 ± 9.022e-6	4.073e-5 ± 1.146e-5	7.606e-5 ± 1.641e-5
Valine	1.004 - 1.035(d)	0.0060 ± 0.0003	0.0046 ± 0.0004 *	0.0054 ± 0.0002
Glutamine	2.45 (m)	0.0166 ± 0.0011	0.0136 ± 0.0013	0.0139 ± 0.0008
Glycine	3.538 - 3.544	0.0068 ± 0.0009	0.0054 ± 0.0010	0.0074 ± 0.0008
Alanine	1.45 (d)	0.0070 ± 0.0004	0.0058 ± 0.0004	0.0064 ± 0.0005
Histidine	7.01 - 7.07 (s)	0.0011 ± 0.0001	0.0006 ± 0.0000 **	0.0007 ± 0.0000
Serine	3.947 - 3.985	0.0038 ± 0.0004	0.0041 ± 0.0005	0.0041 ± 0.0003
**Ketogenesis**				
Acetoacetate/Acetone[a]	2.195 - 2.217 (s)	0.0032 ± 0.0007	0.0033 ± 0.0006	0.0040 ± 0.0006
3-Hydroxybutyrate	1.16 - 1.189 (d)	0.0060 ± 0.0004	0.0024 ± 0.0005 ****	0.0042 ± 0.0005 *, #
3 HB / Acetoacetate		1.1250 ± 0.0892	0.7038 ± 0.0051 ***	1.086 ± 0.0655 ##
**TCA cycle metabolism and intermediates**				
Acetate	1.89 - 1.90 (s)	0.0027 ± 0.0004	0.0022 ± 0.0002	0.0023 ± 0.0003
Lactate	1.292 - 1.33 (d)	0.0800 ± 0.0094	0.0553 ± 0.0055 *	0.0604 ± 0.0048
Succinate	2.379 - 2.387 (s)	0.0024 ± 0.0004	0.0020 ± 0.0003	0.0026 ± 0.0003
Malic acid	4.261 - 4.307	0.0110 ± 0.002	0.0082 ± 0.002	0.0126 ± 0.001
Glucose	5.189 - 5.235	0.0261 ± 0.001	0.0321 ± 0.0001 **	0.0290 ±0.0000
Lactate /Alanine		12.5900 ± 0.8709	9.1790 ± 1.1030	9.8990 ± 1.2950
**Plasma protein**				
N-acetyl-glycoproteins	2.004- 2.045(s)	0.0165 ± 0.0010	0.0127 ± 0.011 *	0.0109 ± 0.0007 **
**Creatine metabolism**				
Creatine	3.005 - 3.023(s)	0.0034 ± 0.0004	0.0031 ± 0.0003	0.0030 ± 0.0004
**Choline metabolism**				
Choline	3.175 - 3.184(s)	0.0055 ± 0.0010	0.0041 ± 0.0008	0.0052 ± 0.0010
Betaine	3.26(s)	1.58e+11 ± 2.38e11	8.68e+10 ± 1.04e10 *	1.40e+11 ± 7.64e11
**Fatty acid metabolism**				
Glycerol	3.64 - 3.635	0.0063 ± 0.0007	0.0049 ± 0.0005	0.0047 ± 0.0002
**Others**				
Unknown (DMSO?)	3.13 (s)	0.0010 ± 0.0001	0.0003 ± 7.98e-5 ****	0.0006 ± 7.89e-5 ***
Mannose	5.149 - 5.176	0.0009 ± 0.000	0.0011 ± 0.0002	0.0010 ± 0.000

Results are expressed as mean ± SEM. n = 8 – 10/group; * $p<0.05$, ** $p<0.01$, *** $p<0.001$, and **** $p<0.0001$ vs. CTRL; # $p<0.05$, ## $p<0.01$ vs. HSuHF.

[a] Putatively annotated as level 3 of identification according to Chemical Analysis Working Group (CAWG) Metabolomics Standards Initiative recommendations. All other metabolites identified as level 2.

Table S3. Metabolites detected in liver samples by ^{1}H-NMR analysis.

Hepatic metabolites	Chemical shift (ppm)	CTRL	HSuHF	HSuHF + BJ
**Amino acids and BCAA metabolism**				
Alanine	1.48 (d)	0.0062 ± 0.0002	0.0058 ± 0.0002	0.0049 ± 0.0001 ****,##
Aspartate	2.80 (m)	0.0007 ± 2.61e-5	0.0008 ± 6.53e-5	0.0007 ± 4.09e-5
Glutamine	2.46 (m)	0.0151 ± 0.0005	0.0159 ± 0.0006	0.0159 ± 0.0006
Glutamate	2.36 (m)	0.0059 ± 0.0005	0.0066 ± 0.0006	0.0051 ± 0.0003
Glycine	3.56 (s)	0.0052 ± 0.0002	0.0046 ± 0.0002	0.0046 ± 0.0001
Histidine	7.13 (s)	0.0002 ± 2.21e-5	0.0002 ± 2.77e-5	0.0002 ± 2.88e-5
Isoleucine	1.01 (d)	0.0012 ± 3.40e-5	0.0009 ± 4.59e-5 ***	0.0010 ± 3.16e-5
Leucine	0.96(d)	0.0017 ± 3.31e-5	0.0016 ± 0.0001	0.0015 ± 3.41e-5
Phenylalanine	7.29-7.45 (m)	0.0012 ± 4.38e-5	0.0010 ± 0.0001	0.0009 ± 2.56e-5
Taurine	3.43 (t)	0.0024 ± 0.0004	0.0026 ± 0.0003	0.0045 ± 0.0005 **, #
Tyrosine	6.91(d),7.18 (d)	0.0004 ± 2.00e-5	0.0003 ± 3.34e-5	0.0003 ± 1.32e-5
Valine	1.05 (d)	0.0019 ± 7.40e-5	0.0015 ± 0.0001 *	0.0014 ± 4.12e-5
**Ketogenesis**				
3-Hydroxibutyrate	1.20 (d)	0.0011 ± 5.90e-5	0.0013 ± 0.0001	0.0014 ± 7.67e-5
**TCA cycle metabolism and intermediates**				
Acetate	1.92 (s)	0.0012 ± 5.58e-5	0.0011 ± 9.77e-5	0.0010 ± 2.97e-5
Lactate	1.33 (d)	0.0196 ± 0.0007	0.0189 ± 0.0009	0.0169 ± 0.0006
Succinate	2.41 (s)	0.0076 ± 0.0003	0.0064 ± 0.0003	0.0061 ± 0.0005
Glucose	5.24 (d)	0.0149 ± 0.0008	0.0182 ± 0.0009 *	0.0163 ± 0.0005
**Creatine metabolism**				
Creatine	3.03 (s)	0.0008 ± 3.73e-5	0.0005 ± 0.0001 *	0.0008 ± 7.66e-5
**Choline metabolism**				
Choline	3.20 (s)	0.022 ± 0.0001	0.0022 ± 0.0008	0.0022 ± 0.0002
Phosphocholine	3.22(s)	0.0152 ±0.0008	0.0092 ± 0.0021	0.0108 ± 0.0034
Glycerolphosphocholine	3.67 (m)	0.0106 ± 0.0004	0.0092 ± 0.0003	0.0089 ± 0.0006
Betaine	3.25 (s)	0.0191 ± 0.0153	0.0076 ± 0.0007 **	0.0164 ± 0.0029 #
**Microbial metabolism**				
TMAO	3.27 (s)	0.0045 ± 0.0007	0.0039 ± 0.0006	0.0068 ±0.0007 #
**Others**				
Glutathione	2.97 (d)	0.0035 ± 0.0014	0.0028 ± 0.0002 *	0.0034 ± 0.0002
Nicotinurate	8.94 (m)	0.0004 ± 2.60e-5	0.0003 ± 3.46e-5	0.0003 ± 4.04e-5
NADP+	9.30 (s)	0.0004 ± 2.20e-5	0.0004 ± 2.59e-5	0.0004 ± 1.53e-5
NAD	9.34 (s)	0.0007 ± 3.10e-5	0.0008 ± 5.04e-5	0.0009 ± 5.20e-5

Results are expressed as mean ± SEM. n = 8 – 10/group; * p<0.05, ** p<0.01, *** p<0.001 and **** p<0.0001 vs. CTRL; # p<0.05, ## p<0.01 vs. HSuHF.
